# Epigenomic Reprogramming of Adult Cardiomyocyte-Derived Cardiac Progenitor Cells

**DOI:** 10.1038/srep17686

**Published:** 2015-12-14

**Authors:** Yiqiang Zhang, Jiang F Zhong, Hongyu Qiu, W. Robb MacLellan, Eduardo Marbán, Charles Wang

**Affiliations:** 1 Division of Cardiology, Department of Medicine, and Center for Cardiovascular Biology, and Institute for Stem Cell and Regenerative Medicine, University of Washington, Seattle, WA 98109, USA; 2 The Heart Institute, Cedars-Sinai Medical Center, Los Angeles, CA, 90048, USA; 3 Ostrow School of Dentistry and Department of Pediatrics, School of Medicine, University of Southern California, Los Angeles, CA 90089, USA; 4 Department of Basic Sciences, School of Medicine, Loma Linda University, Loma Linda, CA 92350, USA; 5 Center for Genomics & Department of Basic Sciences, School of Medicine, Loma Linda University, Loma Linda, CA, 92350, USA

## Abstract

It has been believed that mammalian adult cardiomyocytes (ACMs) are terminally-differentiated and are unable to proliferate. Recently, using a bi-transgenic ACM fate mapping mouse model and an *in vitro* culture system, we demonstrated that adult mouse cardiomyocytes were able to dedifferentiate into cardiac progenitor-like cells (CPCs). However, little is known about the molecular basis of their intrinsic cellular plasticity. Here we integrate single-cell transcriptome and whole-genome DNA methylation analyses to unravel the molecular mechanisms underlying the dedifferentiation and cell cycle reentry of mouse ACMs. Compared to parental cardiomyocytes, dedifferentiated mouse cardiomyocyte-derived CPCs (mCPCs) display epigenomic reprogramming with many differentially-methylated regions, both hypermethylated and hypomethylated, across the entire genome. Correlated well with the methylome, our transcriptomic data showed that the genes encoding cardiac structure and function proteins are remarkably down-regulated in mCPCs, while those for cell cycle, proliferation, and stemness are significantly up-regulated. In addition, implantation of mCPCs into infarcted mouse myocardium improves cardiac function with augmented left ventricular ejection fraction. Our study demonstrates that the cellular plasticity of mammalian cardiomyocytes is the result of a well-orchestrated epigenomic reprogramming and a subsequent global transcriptomic alteration.

Heart muscle cells in lower vertebrates such as zebrafish can be substantially regenerated by dedifferentiation and proliferation of pre-existing cardiomyocytes^1^,^2^. On the other hand, the adult mammalian heart has long been thought to be a non-regenerative organ. This dogma has been challenged by increasing evidence demonstrating that postnatal cardiomyocytes do proliferate at a low rate and contribute to myocardial renewal either physiologically or under stress^3^,^4^,^5^. More controversial is what role, if any, CPCs may play in the injured heart^6^,^7^,^8^. Using a genetic cell fate mapping system and a pure cardiomyocyte culture technique, we recently demonstrated that the mature mammalian cardiomyocytes retained a substantial cellular plasticity. We found that cardiomyocytes can spontaneously dedifferentiate and re-enter into cell cycle in primary cell culture, and subsequently recapture, at least partially, the properties of CPCs^9^. However, the molecular mechanism regulating the spontaneous dedifferentiation of the adult cardiomyocytes into CPCs is not yet understood. It is unknown if there is a genome-wide epigenomic reprograming, e.g., change of the methylome, which results in a transcriptomic alteration in CPCs. In current study, we test the hypothesis that genome-wide epigenomic reprogramming, e.g., change of DNA methylome, underlies the transcriptomic alteration and the spontaneous dedifferentiation of ACMs.

Seemingly in a reversal manner to differentiation, cellular dedifferentiation is the regression of a differentiated, specialized cell or tissue to a primitive state with augmented plasticity. It is a natural mechanism for tissue regeneration and repair, particularly in lower vertebrates^10^,^11^,^12^,^13^. The dedifferentiation process results in remarkable alterations in morphology, function, cellular and molecular features. Dedifferentiation has been characterized at molecular level in fungi, zebrafish and newt hearts, newt lens, and murine myotubes^14^,^15^,^16^,^17^. While cardiomyocytes in primitive animals can dedifferentiate and then regenerate heart muscle, mammalian cardiomyocytes have only been shown to dedifferentiate morphologically in culture and in injured myocardium. Moreover, the molecular characteristics of dedifferentiated cardiomyocytes remain largely undetermined^9^,^18^,^19^,^20^,^21^,^22^,^23^,^24^. Our recent studies demonstrated that adult myocytes can dedifferentiate, re-enter cell cycle, and regain properties of CPCs when cultured for prolonged period. Such dedifferentiated cells can be re-differentiate into cardiomyocytes with spontaneous contractile activity^9^. It has been shown that dedifferentiation occurs prior to the proliferation of neonatal cardiomyocytes in culture^25^. Genetically-labeled proliferating cardiomyocytes were smaller and showed less maturation in injured myocardium^4^,^26^,^27^. Although the mechanisms underlying acquired pluripotency, e.g., induced pluripotent stem cells (iPSCs), have been well studied, the spontaneous dedifferentiation of somatic cells is poorly understood. Cellular dedifferentiation in the induction processes of iPSC is associated with a genome-wide epigenomic reprogramming^28^,^29^. Epigenomics deals with various epigenetic elements and the genomic landscape of stable, yet reprogrammable nuclear changes that control gene expression. DNA methylation is a chief mechanism in the epigenetic modification of gene expression, and it occurs at cytosines of the dinucleotide sequence CpG. Methylation in promoter regions is generally repressive of transcription in the associated genes. It has been shown that both the promoter and non-promoter regions can be regulated by methylation during embryonic development and disease progression^30^,^31^,^32^.

Although all cells in an individual organism or tissue may have a virtually identical genome, each cell has a unique transcriptome that reflects the expression of a subset of genes, which can be affected by epigenetic states. Single-cell transcriptome analysis allows us to access the gene regulatory network at a whole-genome scale to identify genes and pathways that underlie the given cell type’s physiological functions, behavior and phenotype during development^33^. Since dedifferentiation and cellular reprogramming are often asynchronous^34^, it is essential to investigate the transcriptome at single-cell level, which may shed light into the understanding of the underlying molecular mechanisms. Moreover, cell-to-cell variations in gene expression are critical in the development of many tissues^35^,^36^. Although this variation is especially important for stem cell differentiation and cellular dedifferentiation, it has been extremely challenging to measure and interpret data from a single cell in terms of genome-wide transcriptional activity due to random biological variation which, in certain conditions, may not be functionally consequential, in additional to inherent system measurement errors^37^,^38^,^39^.

In this study, we captured freshly-isolated single adult cardiomyocytes (controls) and mCPCs derived from dedifferentiated cardiomyocytes using a microfludic chip coupled with Affymetrix GeneChip for whole-transcriptome analysis to understand the molecular mechanisms regulating adult cardiomyocyte dedifferentiation and reprogramming. Furthermore, using two types of NimbleGen tiling arrays including NimbleGen Mouse DNA Methylation 3x720K CpG Island Plus RefSeq Promoter Array and Comprehensive High-throughput Arrays for Relative Methylation (CHARM)-based DNA methylation analysis techniques for whole-genome DNA methylome analyses, we found that the dedifferentiated mCPCs display epigenomic reprogramming with many differentially-methylated regions, both hypermethylated and hypomethylated across the entire genome which was well correlated with the transcriptomic change. We observed an inverse correlation between global DNA methylation and transcriptomic expression, i.e., at methylome level, many genes were hyper-methylated in the promoter regions with a corresponding down-regulation of their transcripts in mCPCs. Therefore, our results demonstrate an orchestrated genome-wide epigenomic reprogramming and a subsequent transcriptomic change in mCPCs with a molecular signature displaying dedifferentiation, cell cycle reprogramming and acquired stemness.

## Results

### Dedifferentiation of adult cardiomyocytes into mCPCs and the restoration of cardiac function of infarcted mouse hearts after mCPC transplantation

**Figure 1:**
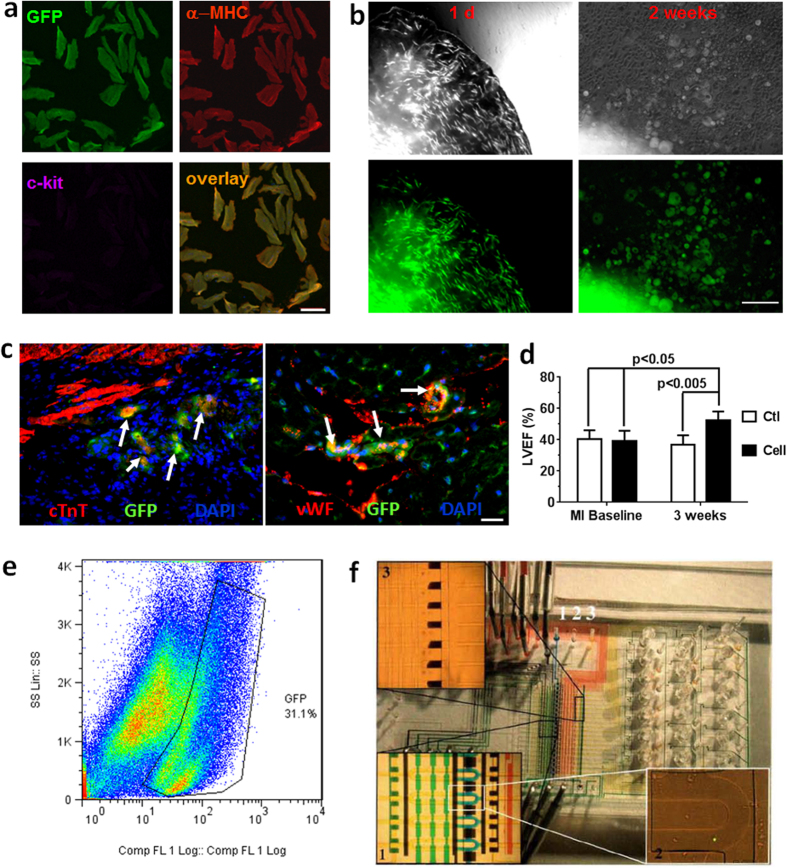
Cardiomyocyte dedifferentiation, re-differentiation, and isolation of single cells for whole-transcriptome analysis. (**a**) Immunocytochemistry showing cardiac-specific labeling of GFP (green) in mature myocytes expressing α-MHC (red) but not c-kit (magenta). Adult cardiomyocytes were isolated from bi-transgenic αMHC-MerCreMer Z/EG mouse hearts after tamoxifen-induced gene recombination (see Methods for detail). Scale bar, 200 μm. (**b**) Phase contrast images (upper panels) and fluorescent images (lower panels, Green- GFP) of transgenic cardiac tissue and derived cells at the beginning of culture (1d) and at 2 weeks of culture. Scale bar, 200 μm. (**c**) Confocal microscope images showing the re-differentiation of GFP-mCPCs (green) into cardiomyocytes expressing cardiac troponin (cTnT, red; white arrows, left panel) and endothelial cells expressing von Willebrand factor (VWF, red; white arrows, right panel) 3 weeks after implantation into the infarcted myocardium of wild-type background mice. Nuclei were stained with DAPI (blue). Scale bar, 100 μm. (**d**) Left ventricular ejection fraction (LVEF) measured at baseline after myocardial infarction (MI) surgery and 3 week post-MI using echocardiography. Vehicle media (Ctl) was used as a control for GFP-mCPC cell transplantation (Cell). N = 5 mice for Ctl group, and 6 mice for Cell group. (**e**) Flow cytometry analysis of cardiac explant cultures at 2 weeks showing about 30% cells are GFP positive in the whole population. (**f**) Custom phase-switch microfluidic chip for the isolation of single mCPCs. Individual live single-cell can be encapsulated into as little as 500-pl droplet for downstream analysis.

Cardiomyocytes from adult heart demonstrated characteristic dedifferentiation features after continuous cultivation in mitogen-rich medium, and gave rise to semi-adherent cells that could subsequently self-organize into cardiospheres capable of spontaneous contraction^9^. Conceivably, cardiomyocytes exhibiting dedifferentiation features could come from pre-existing cardiomyocytes or from incomplete cardiac differentiation (maturation) of progenitor cells. To specifically track cardiomyocytes, we generated MerCreMer/ZEG bi-transgenic mice^5^,^9^,^40^. After Cre-mediated gene recombination induced by tamoxifen treatment, cardiomyocytes were genetically labeled with green fluorescent protein (GFP) (Fig. 1a), enabling cell fate tracking on the GFP-expressing cardiomyocytes and their progenies which remain GFP-positive^5^,^9^,^26^. As shown in Fig. 1b, GFP-cardiomyocytes and other cells migrated off a fragment of genetically-tagged heart tissue in culture. Over time, GFP-positive cardiomyocytes rounded up and lost the expression of cardiac-specific contractile proteins. Notably, ~30% of cells within the culture were GFP^+^-cardiomyocyte-derived cells, and ~20% co-expressed c-kit, a putative cardiac progenitor cell marker with controversial roles (Fig. 1b,e, Supplementary Figs S1 and S2), while Sca-1 was not detected in dedifferentiated cells (data not shown). In bi-transgenic mouse heart subjected to myocardial infarction (MI), there was an increase in c-kit^+^ cells that were also GFP^+^ (originated from ACMs) (Supplementary Fig 3). Furthermore, in an *in vivo* cell transplantation experiment, the GFP mCPC cells were able to differentiate into cardiomyocytes expressing cardiac structure gene Troponin T (cTnT) and endothelial cells expressing von Willebrand factor (VWF) 3 weeks after they were implanted into the infarcted myocardium of wild-type background mice (Fig. 1c). Compared to the vehicle control, cell transplantation also improved cardiac function with augmented left ventricular ejection fraction (LVEF) (Fig. 1d).

### Single-cell transcriptome analysis of mCPCs

To address the heterogeneity of the resultant cell population, we employed a robust microfludic chip system to capture single cells including both adult cardiomyocytes (ACMs) and mCPCs which were then subject to single-cell whole transcriptome analysis (Fig. 1f)^9^,^41^. The adult cardiomyocytes were used as controls. Previous studies have demonstrated the feasibility of generating complementary DNA (cDNA) libraries from single cells^41^,^42^,^43^,^44^,^45^,^46^,^47^,^48^, yet with drawbacks such as 3′ bias or insufficient product for microarray hybridization^44^. In this study, we used the WT-Ovation One-Direct System (NuGen) in which the amplification is initiated at the 3′ end as well as randomly throughout the whole transcript for each gene. This feature, along with highly-refined amplification chemistry, makes the WT Ovation One-Direct System ideal for amplification of the smallest biological samples down to the single-cell level. Amplified cDNAs from single-cells were labeled with biotin and then hybridized with Affymetrix MG 430 2.0 arrays (Supplementary Fig. S4).

**Figure 2:**
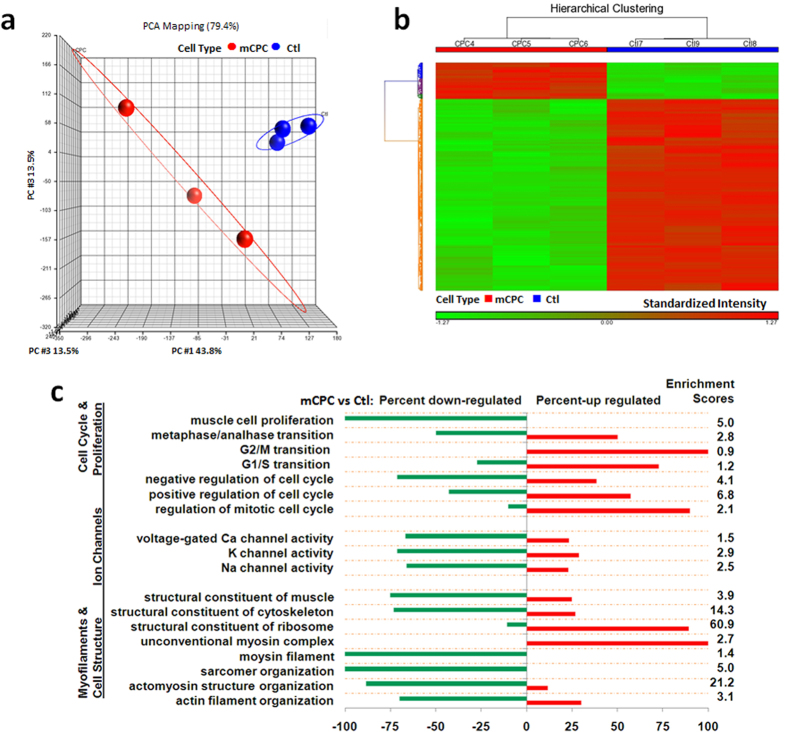
Single-cell transcriptomic differences between parental cardiomyocytes (Ctl) and their derived cardiac progenitor cells (mCPC). (**a**) Principle Component Analysis (PCA) plot of single-cell whole transcriptome profiled by Affymetrix GeneChip microarray. Each blue or red dot represents a single cardiomyocyte (Ctl) or mCPC, respectively. (**b**) Hierarchical clustering of differentially expressed genes with |fold-change| ≥ 1.5 plus P = 0.05; (FDR-adjusted p ≤ 0.05). (**c**) Gene ontology enrichment analysis (GOEA): Percent down- or up-regulated genes passed FDR-adjusted p ≤ 0.05 in the groups of different gene ontology categories, including myofilaments and cellular structure, ion channels, and cell cycle and proliferation were plotted, with Enrichment Scores shown on the right. Ctl: mature cardiomyocytes; mCPC: Mouse cardiomyocyte-derived cardiac progenitor cells.

Principal Component Analysis (PCA) showed that there was a distinct difference in the whole-transcriptome between mCPCs and ACMs (Fig. 2a). Using fold-change (2-fold) plus P ≤ 0.05; false-discovery rate (FDR)-adjusted P value (≤0.05) as threshold, we identified 576 (annotated) differentially-expressed genes (DEGs, Supplemental Dataset). Hierarchical clustering analysis (HCA) showed a clear separation between mCPCs and ACMs based on the DEGs even in a less stringent criteria (1.5-fold plus P = 0.05, FDR ≤ 0.05) (Fig. 2b).

Gene Ontology Enrichment Analysis (GOEA) based on DEGs showed strong functional enrichment (Fisher Exact test, p ≤ 0.05). GOEA revealed that many of these genes were involved in myofilament and cellular structure, and cardiac ion channels; most genes were down-regulated in mCPCs compared to AMCs (Fig. 2c, and Supplementary Table S1). For example, genes related to the specialized cardiac functions were largely down-regulated in mCPCs compared to AMCs. Among these genes, ryanodine receptor, a critical molecule governing cardiac excitation-contraction coupling and metabolism, decreased >100-fold in mCPCs. Consistently, both α- and β-myosin heavy chain (MHC) genes *myh6* and *myh7*, cardiac troponin T and I (*Tnnt2*, *Tnni3*), and Titin (*Ttn*) were down-regulated remarkably in mCPCs, whereas the putative proliferative *ether-à-go-go-related* (EAG) potassium channel gene *Kcnh3* was up-regulated in mCPCs^49^,^50^. Cardiac ion channel genes that typically expressed in normal adult myocytes were decreased significantly in mCPCs, along with parallelly augmented expression of inhibitory accessory units such as *Kcne4* (encoding MirP3)^51^. GOEA also revealed the reprogramming of cell cycle genes in mCPCs, signified by a down-regulation of genes known to suppress muscle cell proliferation and by an up-regulation of genes promoting cell cycle progression (Fig. 2c, and Supplementary Table S2). Cell cycle-related genes, such as cyclin D2 (*Ccnd2*, 5.5-fold), cyclin-dependent kinase 4 (*Cdk4*, 8-fold) and CDC28 protein kinase regulatory subunit 2 (*Cks2*, 47.8-fold) were up-regulated in mCPCs, with an oscillating decrease of CDK inhibitor 1B (*Cdkn1b*) that transcribes the negative cell cycle protein p27^52^,^53^. Furthermore, other cell proliferation-related genes, such as *Ereg* (epiregulin, 37.7-fold), *Myc* (myelocytomatosis oncogene, 32.3-fold) and *Tgfβ2* (β2-transforming growth factor, 6.0-fold) were also up-regulated significantly (Supplemental Table S3). Notably, Tgfβ2 is also a multifunctional cytokine involved in regulating somatic stem cell division (Supplementary Table S4)^54^.

Ingenuity Pathway Analysis (IPA) further suggested that these differentially-expressed genes were involved in multiple functional networks, such as tissue morphology, muscular system development, and metabolism, as well as cell cycle and development functions (Supplementary Information). The top ranked genes, such as *Myh6* (−170-fold), *Pln* (−171.5-fold), *Tnni3* (−130.7-fold), *Ttn* (−74.2-fold), *Mef2c* (−19.9-fold), and *Actc1*(−112.4-fold) were remarkably down-regulated in mCPCs, whereas *Fosl1* (+39.4-fold) and *Myc* (+32.3-fold) were up-regulated in mCPCs as compared to AMCs, underscoring the dedifferentiation and cell cycle reprogramming in mCPCs. Myc, Cdkn1a (p21) and Gsk3b were central hubs among these regulated molecules. These signaling pathways may be interventional points for future studies on dedifferentiation, cell cycle reprogramming and cell fate transformation (Supplementary Fig. S5).

### Validation of gene expression by Real-Time qPCR and TLDA at single-cell level

**Figure 3:**
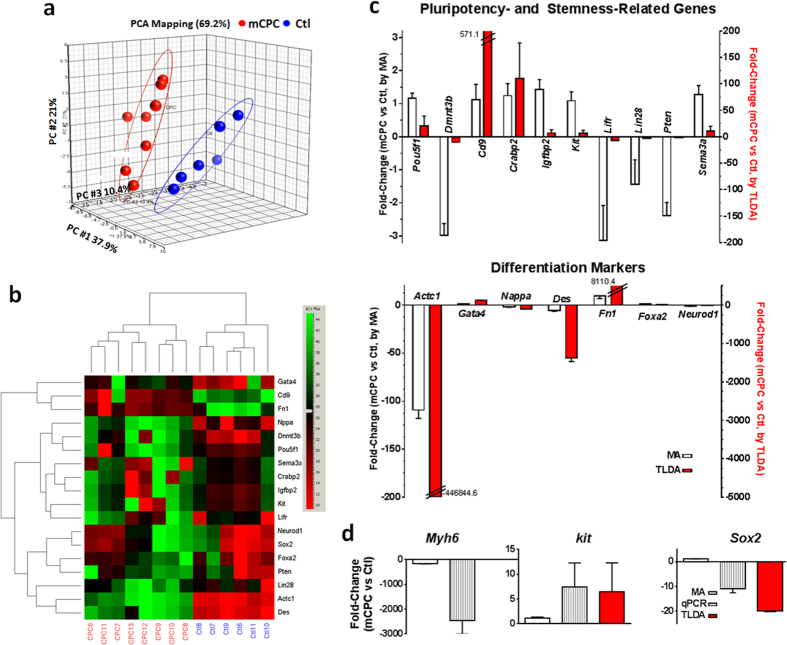
Validation of microarray (MA) data by TaqMan low density array (TLDA) and by single-cell TaqMan qPCR assays (qPCR). (**a**) PCA plot showing the separation of cardiomyocytes (Ctl, blue) from mouse cardiomyocyte-derived cardiac progenitor cells (mCPCs, red) in their gene expressions as detected by TLDA. (**b**) Hierarchical clustering of TLDA genes detected in either adult cardiomyocytes (Ctl) or their derived mCPCs with differential expression. (**c**) Expression level of genes for pluripotency and stemness-related (top panel) and differentiation markers (lower panel) detected by TLDA. For TLDA, the cDNA from each single-cell (6 control adult cardiomyocytes and 8 mCPCs, biological replicates) was run. Normalized probeset intensity exported from Affymetrix Gene Console was used for the calculation of gene expression fold changes detected microarray for 3 single cardiomyocytes and 3 single mCPCs. (**d**) Expression levels of cardiomyocyte gene *Myh6*, cardiac progenitor gene *c-kit*, and stem cell gene S*ox2* in mCPCs as compared to control cardiomyocytes. N = 5 single cells (mCPCs or adult control cardiomyocytes; technically triplicated for each cell). Note that the preconfigured pluripotency TLDA did not include *Myh6* gene.

We verified the expression levels of a number of genes in single cells by either TaqMan Real-Time qPCR (3 genes) or pluripotency/differentiation TaqMan Low Density Array (TLDA, 96 genes). To circumvent the detection limit of qPCR, a pre-amplification step was performed after synthesis of cDNA from each single-cell lysate. Pre-amplification remarkably reduced the threshold cycle value (~10 Ct) for the housekeeping gene *Gapdh* (data not shown), therefore providing sufficient cDNA for transcript detection. HCA analysis on a panel of stem cell/pluripotency and differentiation genes detected by TLDA using single-cell cDNAs revealed a clear segregation between mCPCs and parental ACMs (Fig. 3a,b). Furthermore, single-cell level TLDA showed that the genes for stem cell pluripotency were mostly not detected in mature cardiomyocytes, but many of their expressions were elevated in mCPCs. It is clear that these mCPCs did not express all the stemness genes found in embryonic stem cells or induced pluripotent stem cells, and only 52 genes among the full panel had a Ct value lower than 40. Compared with single-cell microarray (MA) transcriptomic data, single-cell TLDA data showed that mRNA expression levels for most pluripotency and stemness-related genes and differentiation markers were very similar or exhibited similar trends (Fig. 3c). For instance, Oct4 (*Pou5f1*) gene was up-regulated in mCPCs, while most differentiation markers (e.g., *Actc1*, *Des*) were down-regulated, detected by both MA and TLDA. Furthermore, our single-cell level qRT-PCR also confirmed the single-cell microarray results, e.g., there was a significant decrease in *Myh6* (−157.6 folds by MA, −2463.7 folds by qPCR; note that *Myh6* is not included in the preconfigured TLDA), but an increased gene expression in *c-kit* (7.4 folds by single-cell TaqMan qPCR; 6.5 folds by TLDA qPCR, though no significant change detected by MA); nevertheless *Sox2* was intriguingly down-regulated in mCPCs (−10.9 folds by single-cell TaqMan qPCR, and −20.0 folds by TLDA qPCR, but no significant change by MA (1.1 fold)) (Fig. 3d). The presence of *Sox2* transcript in cardiomyocytes was unprecedented but was consistently detectable by both single-cell TaqMan RT-qPCR and single-cell TLDA assays. With the state-of-the-art technologies applied in the current study and the consistent convincing results, we confirmed at a single-cell whole-transcriptomic level the significant molecular signatures featuring the dedifferentiation and cell cycle reprogramming in mCPCs derived from adult cardiomyocytes.

### Epigenetic reprogramming of mCPCs

**Figure 4:**
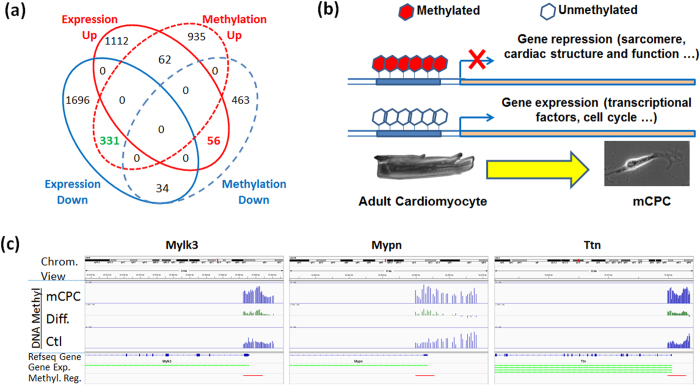
Epigenetic regulation of transcriptional changes in mCPC cells. (**a**) Venn diagram of overlapping differentially-expressed genes (|Fold change| ≥ 1.5 plus P = 0.05, FDR-adjusted p ≤ 0.25; Affymetrix mouse GeneChip 430 2.0) and differentially-methylated genes (defined as p ≤ 0.05; combination of the CHARM analysis on the data from NimbleGen 3 × 720 k Promoter array and customer 2.1 M CHARM array). (**b**) Model depicting the epigenetic regulation in mCPCs derived from dedifferentiated cardiomyocytes. The promoters of adult and cardiac specific genes, and sarcomere structure genes become hypermethylated, resulting in their transcriptional repression in mCPCs, while transcriptional factors important to cell cycle/proliferation and progenitor stemness are not or less methylated, conferring their expression in mCPCs. (**c**) Visualization of the methylation in promoter regions of sarcomere genes myosin light chain kinase 3 (*Mylk3*), myopalladin (*Mypn*), and Titin (*Ttn*) detected by the NimbleGen 3 × 720 k DNA methylation promoter array. Top panel shows the chromosomal region flanking these genes. Middle panel: the middle bar graphs present the DNA methylation levels (1.0 = 100%) in mCPCs and control cardiomyocytes (Ctl), as well as the differential methylation level (Diff.) comparing mCPC to Ctl. The bottom portion shows the Refseq gene structure, the expression of transcript detected by probe(s) for which green color denotes down-regulation, and the differentially-methylated region in mCPCs vs. cardiomyocytes.

Changes in regional DNA methylation underlie the plasticity of progenitors and stem cells, and have been implicated in the epigenetic reprogramming of iPSCs derived from somatic cells^28^,^29^. We used two different Roche NimbleGen tiling arrays (3 × 720 k and CHARM 2.1 M designs) with maximal genome coverage and cross-validation each other to interrogate genome-wide DNA methylation patterns. We found that overall, there was a great differential methylation between mCPC and AMC and this observation was consistent in two different NimbleGen arrays (Supplementary Fig. S6). Furthermore, the gene expression at transcriptomic level was well orchestrated with DNA methylation in mCPCs, i.e., there were substantially more down-regulated genes than up-regulated genes in the transcriptome, whereas there were more regions of hyper-methylation than regions of hypo-methylation. There was an inverse correlation between DNA methylation at promoters of CpG island regions and the transcript expression for many genes, i.e., while their relative methylation levels were elevated, their transcriptional expressions were repressed in mCPC (Fig. 4a,b, and Supplemental datasets).

**Figure 5:**
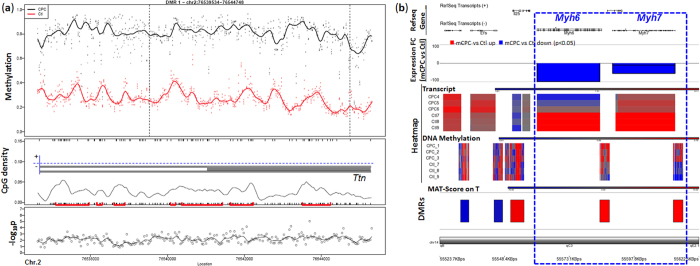
Genomic view displaying DNA hyermethylation of *Ttn*, *Myh6* and *Myh7* in mCPCs. (**a**) Differentially-methylated regions (DMRs) in a portion of the Titin (*Ttn*) gene detected by CHARM customer array. DMRs were located in both CpG islands and shores (with, or without red marks in the middle panel, respectively). Overlaid onto the CpG density plot is the *Ttn* gene structure –*Ttn* is on the minus strand, and the gray box represents exon. The upper panel shows the methylation level (1.0 = 100%) of adult cardiomyocytes (Ctl, red) and their derived cardiac progenitor cells (mCPCs, black). The vertical dash lines define the edges of the DMR. The lower panel shows the chromosome locations and differential methylation p values for probes. (**b**) Genomic view showing differential DNA methylation and differential gene expression between mCPCs and control cardiomyocytes. The gene expression of *Myh6* and *Myh7* (dash line boxed) genes was down-regulated in mCPCs compared to control cardiomyocytes, whereas their promoter regions were hypermethylated. The top panel shows the Refseq transcript structure; and note that both *Myh6* and *Myh7* are on the minus strand. Second panel: gene expression fold change (FC) between mCPCs and cardiomyocytes detected by Affymetrix GeneChip gene expression array. The third and fourth panels: transcript and DNA methylation heatmaps. The fifth panel: DMR based on MAT-score on T detected by Roche 3 × 720 k DNA methylation array. The bottom line shows the genome location.

**Figure 6:**
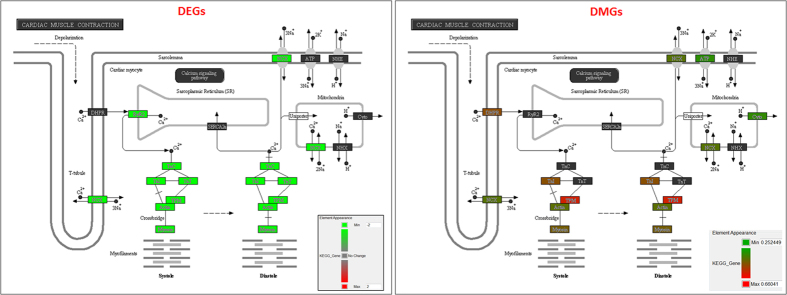
Integrated pathway enrichment analysis based on differentially expressed genes (DEGs) and differentially methylated genes (DMGs). The “cardiac muscle contraction pathway” is enriched by both DEGs and DMGs based on KEGG knowledge database using the DEGs detected by Affymetrix GeneChIP and the DMGs detected by CHARM 2.1M arrays (p ≤ 0.05). The down-regulation of most genes in this pathway (*left*, green color shaded genes) in mCPCs vs. adult cardiomyocytes is well orchestrated with their DNA hypermethylation (*right*, genes annotated with non-black colors have relative methylation level ≥25% in mCPCs vs control cardiomyocytes). The enrichment p value for Cardiac Muscle Contraction pathway is 5.452 × 10^−9^ for gene expression, and 0.000249 for DNA methylation.

Consistent with the gene expression GO enrichment results, our GO enrichment analysis based on differentially methylated genes (DMGs) and/or differentially methylated regions (DMRs) revealed that many cardiac structure genes, such as sarcomere organization (*Mylk3*, *Mypn*, *Tcap*, and *Ttn*; p-value = 7.2 × 10^−8^) and muscle structural component (*Myl3*, *Tcap*, *Ttn*; p-value = 0.00031), displayed an elevated DNA methylation in the promoters/CpG islands and a repressed gene expression. Three out of four sarcomere genes, i.e., *Mylk3*, *Mypn*, and *Ttn*, were shown to have remarkably elevated DNA methylation in the promoter regions (Fig. 4c), whereas for the fourth gene, *Tcap*, the increased methylation was found in the regions located near the downstream of the transcription start site (TSS; Supplementary Fig. S6). Actually, GO category of sarcomere organization had the 3^rd^ lowest p-value, immediately ranked after two other related cardiac structure categories, i.e., Z-disc (*Csrp3*, *Homer1*, *Ldb3*, *Murc*, *Mypn*, *Tcap*, *Ttn*; p-value = 1.4 × 10^−10^) and muscle filament sliding (*Myl3*, *Tcap*, *Tnnc1*, *Tnni3*, *Tnnt2*, and *Ttn*; p-value = 1.4 × 10^−9^) (Figs 4b,c and 5). We also found that hypermethylated regions for some genes, e.g., Titin gene (*Ttn*), were located at both CpG island and shore areas, covering both promoter and gene body regions, leading to its suppression in mCPCs compared to control cardiomyocytes (Figs 4c and 5a, and Supplementary Fig. S7). Additionally, both *myh6* and *mhy7* were hypermethylated in mCPCs, while re-expressed *Ereg* and *Sox4* were hypomethylated (Fig. 5b, and Supplementary Figs. S8–9). To examine if DEGs and DMGs overlap in their signaling pathways and networks, Pathway Enrichment Analysis was carried out using DEGs and DMGs based on the KEGG (Kyoto Encyclopedia of Genes and Genomes) pathway database. The top ranked pathways converged by both DEGs and DMGs were highly consistent, including those of cardiac muscle contraction, hypertrophic response, oxidative phosphorylation and metabolism, with the methylation status reversely correlated to the transcript expression (Fig. 6, and Supplementary Fig. S10). Together, this indicates that development of muscle contraction in differentiated cells is associated with the loss of methylation in the promoters of these genes^55^,^56^. However, in dedifferentiated cells derived from mature cardiomyocytes, hypermethylation leads to their transcriptional repression.

## Discussion

Cell fate was generally believed to be unidirectional and irreversible although the concept and phenomena of cellular dedifferentiation had been documented for a centry^57^. Adult mammalian cardiomyocytes have traditionally been viewed as terminally-differentiated cells unable to divide. However, mounting evidence now supports the notion that functionally-specialized cells, ranging from plant to mammalian, can change their fate under the influence of environmental factors. Protoplasts from tobacco leaves undergo a dedifferentiated phase conferring pluripotency that precedes signal-dependent re-entry into the cell cycle^58^. Human chondrocytes, epidermal cells, pancreatic beta cells and adipose stromal cells dedifferentiate and exhibit progenitor cell phenotypes and functions^59^,^60^,^61^,^62^,^63^. Cardiomyocyte dedifferentiation has been reported as an undesirable phenotypic change in previous studies by electrophysiologists who sought to maintain the adult electrophysiological phenotype of isolated cardiomyocytes^64^. While previous studies have characterized the morphological, electrophysiological and general phenotypic alterations in dedifferentiated cardiomyocytes, little is known about the molecular reprogramming^18^,^20^,^21^,^22^,^23^,^24^,^26^,^65^,^66^. Using lineage tracing specific for adult cardiomyocytes, we demonstrated that the GFP^+^ cardiomyocytes can undergo dedifferentiation (Fig. 1) and express c-kit, a marker previously thought to be expressed in putative CPCs^9^,^67^,^68^. C-kit-positive cells have been demonstrated to be important for proper cardiac development and reach a maximal level in the early postnatal heart^69^,^70^,^71^, while studies using fate mapping of their potential contribution to myocardial regeneration revealed a minimal role for cardiogenesis in normal adult hearts^72^. An increase of c-kit cells in injured myocardium coincides with the augmentation of dedifferentiated cardiomyocytes (Supplementary Fig. S3)^19^,^20^,^26^,^27^. Therefore our data suggest that c-kit may be a better marker for dedifferentiated cardiomyocytes that may reach a state resembling CPCs; such cells may re-differentiate when implanted into infarcted myocardium (Fig. 1) or when organized into spheroid structures^9^. Although long-term dedifferentiation of cardiomyocytes as found in hibernating myocardium or in infarcted hearts may be undesirable for proper cardiac function^19^,^20^,^27^,^73^, dedifferentiation such as disassembly of sarcomeres is necessary for cell division in both zebrafish and rodent cardiomyocytes^1^,^12^,^25^,^74^. Furthermore, dedifferentiated cardiomyocytes are not apoptotic and presumably reflect adaptations to abnormal myocardial stress^19^,^75^. Therefore, understanding the molecular reprogramming of cardiomyocyte dedifferentiation and proliferation and the derived mCPC-like cells may help to formulate strategies for prevention of heart failure and enhancement of cardiac regeneration^68^.

DNA methylation is a major epigenetic mechanism controlling gene expression. The promoter hypermethylation is commonly found in cancers, which induces the transcriptional repression of important growth regulators including tumor suppressor genes. On the other hand, somatic cells such as skin fibroblasts and cardiomyocytes can be reprogrammed and dedifferentiated into iPSCs by defined factors^28^,^76^. Such transformed cells with cellular phenotypes and transcriptomic profiles distinct from their parental cells present substantial epigenetic reprogramming in which stemness genes are hypomethylated. De-methylation in promoter regions of pluripotency factors results in their increased expression during iPSC formation^77^. Hypo-methylated stem cell-specific regions are more likely to present in the CpG islands, whereas the hyper-methylated stem cell-specific regions are likely to occur in the non-CpG islands. This suggests that the promoters in CpG islands for stemness genes have a propensity to be demethylated during reprogramming towards pluripotent stem cells^78^. It was reported that human epithelial cells with a repression of the p16/pRb pathway, similar to what is in stem cells and many tumor cells, undergo epithelial–mesenchymal transition (EMT) with a remarkable epigenetic remodeling, including DNA methylation of the genes silenced in basal-like breast cancers. Consistent with the observation of the direct conversion of fibroblasts into other functional cell types such as neuronal cells or cardiomyocytes which were accompanied with a hypomethylation of the genes specific to neuronal or cardiac cells^79^,^80^,^81^, our current study showed that the cardiomyocyte-specific genes which were hypomethylated in ACMs became substantially hypermethylated in dedifferentiated cells, i.e., mCPCs. It is noteworthy that during cardiac maturation, there is a switch of cardiac myosin heavy chain expression from fetal βMHC isoform (encode by *Myh7*) into adult αMHC isoform (encode by *Myh6*), while αMHC is down- regulated in hypertrophic heart failure^82^. Such a developmental and pathological remodeling in contractile gene expression is associated with transcriptional regulations by histone modifications^83^,^84^. Thus, DNA methylome reprogramming is an additional and common process during cellular transformation, for example, dedifferentiation.

Our results demonstrated that the transcriptomic alteration featuring the loss of mature cardiac molecular properties in mCPCs is a result of epigenomic reprogramming of DNA methylome which leads to a repression in cardiac genes but an up-regulation of cell cycle and proliferation genes. Given the small number of genes showing clear epigenetic changes, it is possible that there may be a more dominant regulator controlling the transcriptional differences between mCPCs and adult cardiomyocytes. To look into this possibility, we used IPA to predict transcription factors with downstream target showing differential gene expression. We observed a plethora of transcription factors that are predicted to regulate the transcriptional changes between mCPC and parental cardiomyocytes. When focusing on the genes showing the strongest differential expression, we could narrow down the list of putative transcription factors that control the augmented genes in mCPCs. We also found that there are a few dozen transcription factors that are predicted to regulate the genes that are down-regulated in mCPCs compared to ACMs (Supplemental Dataset).

Interestingly, many of the transcription factors predicted to cause an up-regulation on the gene are implicated in many important developmental pathways, such as Smad1, Smad2, Smad3, Smad4, Notch1^85^,^86^,^87^,^88^. The significantly upregulated SMADs and Notch1 in mCPCs are critical in embryonic cardiac development and the generation of cardiac progenitor cells, cell survival and cell cycle reprogramming^86^,^87^,^89^. Furthermore, transcription factors that are predicted to be inhibited in mCPCs are likely important in the function of differentiated cells (adult cardiomyocytes), such as myogenic differentiation 1 (Myod1) and myocardin (Myod).

Cardiac-derived stem cells have recently been evaluated in clinical trials for their potential of healing the injured heart after myocardial infarction^90^,^91^. The identification of such clinically-promising cells was primarily based on their phenotypic properties, with little information on their molecular properties. Compared to bone marrow or mesenchymal c-kit cells, cardiac c-kit cells represent the most primitive population in the mouse heart^92^. However, the roles of c-kit cells for myocardial regeneration in adult hearts remained controversial and uncertain even though it was identified as a putative CPC marker for cardiac development^69^,^71^,^72^,^93^,^94^,^95^. Our study has shed light into the molecular reprogramming of adult cardiomyocyte dedifferentiation. Using a microfluidic chip customizable for different cell types, we can capture single cells with minimal interference. The linear cDNA amplification strategy using 3′ initiated and random primers in this study was demonstrated to suffice for single-cell whole-genome analysis. Moreover, with pre-amplification of target genes, it is feasible to validate multiple genes of interest using qPCR at single-cell level. RNA-seq single-cell transcriptome analysis using deep sequencing technique is likely to reveal more subtle changes which are limited by microarray technology^96^,^97^,^98^,^99^,^100^,^101^. We also showed that the GFP mCPCs can redifferentiate into cells of the heart compartment and express cardiomyocyte and endothelial cell markers. However, without additional fluorescent cardiac lineage reporter system which is specific for cardiac nuclei, we are limited in isolating live GFP cells featuring cardiomyocyte phenotypes to analyze their transcriptome and DNA methylome after the re-differentiation of GFP mCPCs. Given that epigenomic reprogramming is one of the critical regulatory mechanisms in induced pluripotency in stem cells^28^, logical future studies would entail examining the epigenetic intervention on cardiomyocytes and progenitor cells to promote cardiac regeneration.

In summary, our study demonstrates a remarkable gene expression change across the mouse genome in cardiac progenitor cells derived from the dedifferentiation of mature cardiomyocytes. The large differential gene expression in mCPCs occurred in corresponding to an epigenomic reprogramming, i.e., a comprehensive genome-wide alteration in DNA methylation. The repression of mature and cardiac specific genes was due to the hypermethylation of their genomic DNA, resulting in the suppression of a number of genes thereby the dedifferentiation and cell cycle reprogramming in mCPCs.

## Materials and Methods

### Bi-transgenic mouse model and modified cardiac explant culture

All experimental protocols were approved by the Institutional Biosafety Committee and Animal Care and Use Committees at Cedars-Sinai Medical Center. The methods were carried out in accordance with the approved guidelines. Bi-transgenic MerCreMer/ZEG mice were produced by crossbreeding the cardiomyocyte-specific αMHC-MerCreMer mice and the ZEG reporter mice (Jackson Laboratory) as described previously^9^,^40^,^102^,^103^. To induce *Cre*-mediated gene recombination for GFP labeling specifically in cardiomyocytes, genotype-verified double heterozygous MerCreMer-Z/EG mice (2-month old) were treated with 4-OH-tamoxifen for 2 weeks followed by a waiting period of 2 weeks as described previously^9^,^26^. Cardiomyocytes were isolated using enzymatic dissociation method as described^9^,^26^. To maximize the viability of transgenic mouse cardiomyocytes and follow their dedifferentiation, modified cardiac explant culture techniques described previously were used to generate mouse cardiomyocyte-derived progenitor cells (mCPCs)^9^,^104^. Briefly, tamoxifen-treated bitransgenic mouse hearts were partially digested in calcium-free Tyrode’s physiological solution supplemented with 0.15 Wünsch unit/mL of collagenase made from Liberase Blendzyme 4 (Roche Applied Science), followed by washing for 3 min in KB solution. The hearts were cut into small pieces in ~0.1 mm^3^ and rinsed in KB solution for 3 times. Tissues were transferred to laminin-coated 10 mm tissue culture dishes, with M199 medium containing 100 U/mL penicillin, 100 μg/mL streptomycin, 5% FBS (Invitrogen), 25 μM Blebbistatin, ITS (5 μg/ml insulin and transferrin, 5 ng/ml selenium), and 10 mM β-hydroxybutyric acid for the first two days of culture. Blebbistatin, ITS, and β-hydroxybutyric acid were replaced with bFGF 0.1 ng/ml and TGF-β3 1 ng/ml, and FBS increased to 20% in subsequent cell culture. Medium was partially replaced every 2–3 days. Loosely adherent mCPCs were harvested by gentle pipetting 3 times with a disposable transfer pipette. Flow cytometry was used to characterize the immuno phenotypes of cardiac explant-derived cells as described previously^105^.

### mCPC cell transplantation and cardiomyocyte dedifferentiation in mouse myocardial infarction model

Acute myocardial infarction (MI) was made in adult female wildtype B6129SF/J mice by permanent ligation of the left anterior descending coronary artery (LAD) as described previously^26^,^106^. Immediately after LAD ligation with 7-0 prolene, hearts were intramyocardially injected with a total of 21 μl of either vehicle (PBS, Control group, N = 5 mice) or 100,000 GFP-positive mCPCs isolated from cardiac explant culture of bi-transgenic mouse hearts (Cell group, N = 6 mice) at 3 points in the border zone of infarct area. Transthoracic echocardiography was performed in mice 4 hours (baseline) and 3 week post-MI using a Vevo 770 imaging system (VisualSonic, Toronto, Ontario, Canada). Left ventricular ejection fraction (LVEF) was measured based on the 2D images in long axis view at the level of the greatest LV diameter. The re-differentiation of mCPCs was assessed by fluorescent immunohistochemistry detecting the co-expression of GFP with cardiac troponin T (cTnT) for cardiomyocyte marker or von Willebrand factor (VWF) for endothelial cell marker^26^,^107^.

To examine the dedifferentiation of cardiomyocytes *in vivo*, LAD ligation-induced MI model was created in tamoxifen-treated bitransgenic MerCreMer-Z/EG mice at 10 weeks of age. Sham control animals underwent the same procedures except for the coronary artery ligation. Mice were euthanized 3 weeks after surgery and hearts were collected for histological studies.

### Microfluidic capture of single cardiomyocytes and cardiac progenitor cells

Freshly isolated adult (~3-month old) cardiomyocytes and mCPCs (derived from GFP-cardiomyocytes) were captured using microfludic chip (Fig. 1f)^41^,^108^. The adult cardiomyocytes were used as controls. Briefly, isolated adult cardiomyocytes were preserved in EGTA-free KB solution^9^, while mCPCs were kept in cold PBS during microfludic separation. Single-cells were isolated by encapsulation in droplet using a microfluidic device as described previously^108^,^109^. Once individual cells were isolated and verified under microscope, each was lysed with 2 μl of a customized buffer for whole genomic amplification for transcriptomic profiling. Alternatively, 5 μl Cell-to-Ct lysis buffer supplemented with DNase I (Ambion) was used to lyse each single cell for the validation of gene expression by Real-Time TaqMan single qPCR assays or by TLDA qPCR array.

### cDNA synthesis and amplification from single cardiomyocytes and cardiac progenitor cells

For microarray study, first strand cDNA was synthesized from single cell lysate using a unique first strand DNA/RNA chimeric primer mix and reverse transcriptase provided by NuGEN Technologies. The primers had a DNA portion that hybridizes either to the 5′ portion of the poly (A) sequence or randomly across the transcript. Reverse-transcription extended the 3′ DNA end of each primer generating first strand cDNA. The resulting cDNA/mRNA hybrid molecule contained a unique RNA sequence at the 5′ end of the cDNA strand and subsequently, double stranded cDNA with DNA/RNA heteroduplex at one end was generated. Ribo-SPIA amplification of cDNA produced enough amount of cDNA for labeling and microarray hybridization.

### cDNA biotin labeling, microarray hybridization and data processing for transcriptomic profiling

For each single cell, 5 μg amplified cDNA was fragmented and used for biotin labeling with NuGen’s Encore Biotin Module. The biotin labeled cDNA was hybridized for 40 hours with Affymetrix Mouse Genome 430 2.0 array. After the hybridization, the chips were washed with GeneChip Fluidics Station 450 (Affymetrix) according to the standard fluidic protocol EUKGE-WS2V5_450 (Affymetrix). Microarray images were acquired and processed using GeneChip Scanner 3000 7G (Affymetrix) and gene expression values were analyzed using Affymetrix Gene Expression Console^97^,^98^.

### Validation of gene expression by TaqMan RT-qPCR and TLDA at single-cell level

cDNA synthesis from individual cell of additional single cardiomyocytes and mCPCs was performed using Cell-to-Ct protocol with pre-amplification option (Ambion). Cardiomyocyte-specific gene (*Myh6*) and putative progenitor and stem cell genes (*c-kit* and *Sox2*) together with two endogenous controls (*Actb* and *Gapdh*) were validated by TaqMan RT-qPCR assays (Applied Biosystems) at single-cell level. Moreover, a panel of genes for stem cell pluripotency and differentiation covered by TaqMan Low Density Array (TLDA, 96 genes including house-keeping genes such as Gapdh, Applied Biosystems) were profiled in a separated subset of single cells using our optimized streamline protocols including single-cell lysis, cDNA synthesis, and pre-amplification. Real-time qPCR was performed on a 7900HT Fast Real-Time PCR System (Applied Biosystems) and data collected and analyzed using SDS 2.3 software suite. Ct values were normalized to endogenous controls, and comparative 2^−ΔΔCt^ method was used to evaluate the relative gene expression in mCPCs *vs*. cardiomyocytes^9^. DataAssist 2.0 (Applied Biosystems) was used to analyze the expression changes. As for comparison, normalized intensity of probe(s) of specific genes was used to calculate gene expression fold changes detected by microarray.

### DNA methylation analysis

Genomic DNA was isolated from population adult cardiomyocytes and population mCPCs using Qiagen AllPrep DNA/RNA Micro Kit. Ten (10) ng of genomic DNA was first subject to a whole genome amplification using Sigma’s GenomePlex Complete Whole Genome Amplification (WGA2) kit. The amplified DNA products were used for whole-genome DNA methylome analyses by microarrays using either NimbleGen Mouse 2.1M Array or NimbleGen Mouse 3x720K CpG Islands Pus RefSeq Promoter Array Tiling Array. Modified standard CHARM protocol with reduced starting amount of amplified genomic DNA (2.5 μg) triplicated in each group was used for two types of arrays^110^,^111^. Restriction enzyme McrBC was used to digest amplified genomic DNA as it recognizes the site (A/G)^m^C(N_40–3000_)(A/G) ^m^C, with an optimal separation of 55–103bp, covering nearly half of all possible 5-methylcytosine nucleotides in the genome^112^. For each sample, half of the amplified genomic DNA (2.5 μg) with sizes ranging 100-1000 bp was subjected to McrBC digestion thereby methylated cytosines were cut into smaller fragments. The other half of amplified genomic DNA (2.5 μg) was not treated with McrBC enzyme. Both McrBC-treated and untreated portions were fractionated by 1% agarose gel. The McrBC-treated portion was methyl-depleted (MD) DNA and the untreated (UT) portion represented the total genomic DNA input. Amplified MD (equivalent to experimental sample) and UT (equivalent to input/control sample) samples were labeled with Cy5 and Cy3, respectively, according to standard NimbleGen Array protocol. Labeled DNA samples were hybridized to either types of arrays according to NimbleGen Array User Guide, and the arrays were scanned using MS 200 Microarray Scanner (Roche NimbleGen) and features extracted by NimbleScan software.

Relative DNA Methylation in cardiomyocytes and mCPCs was analyzed using Roche NimbleGen 2.1 M mouse genome CHARM 2.1M array and NimbleGen DNA Methylation CpG Islands Pus RefSeq Promoter array (3 × 720K). Labeled DNA samples were hybridized to arrays according to NimbleGen Array User Guide, and arrays scanned using MS 200 Microarray Scanner (Roche NimbleGen) and features extracted by NimbleScan software.

### Microarray and TLDA qPCR data analysis

Gene expression data analysis was performed using Partek Genomics Suite (Version 6.6; Partek, Inc., St. Louis, MO) as described previously^96^,^97^,^98^,^99^,^100^. Gene expression values were RMA normalized^113^. Fold-change values were calculated based on the least-squares mean, and FDR values were calculated using the method of Benjamini and Hochberg (based on the distribution of p-values calculated via 1-way ANOVA)^114^. In the analysis for visualization, gene ontology enrichment, and DNA methylation integration, a probe was defined as differentially expressed if it showed a |fold-change| ≥ 1.5 and FDR-adjusted p ≤ 0.25. If a functional group has a GO enrichment score over 1, the functional category is overexpressed; a value of 3 or greater corresponds to a p value of 0.05 or less. The greater the score, the more differentially expressed in the functional group of genes. However, in the transcription factor analysis using IPA (Ingenuity® Systems), slightly more stringent criteria were utilized (|fold-change| ≥ 1.5 and FDR ≤ 0.05) due to the large number of predicted transcriptional regulators. Alternatively, enriched signaling pathways by these significant genes were visualized using GeneSpring GX11 software (Agilent). TLDA data were analyzed using DataAssist 2.0 (Applied Biosystems) and Partek Genomics Suite.

DNA methylation data analysis was conducted using the dmrFind function in the CHARM package in R^110^. Regions were defined as differentially methylated if they showed an unadjusted p-value ≤ 0.05 (with a predicted FDR of 0.077 for the 3 × 720 k array and 0.10 for the custom 2.1 M CHARM array). Genes were defined as differentially methylated if a CHARM peak was within 2000 bases pairs upstream and/or 500 base pairs downstream of the transcription start site (for any transcript described within RefSeq)^115^. 3 × 720 k arrays were also analyzed using Partek Genomic Suite with Tiling Array module: Pair files were imported and Loess normalization was performed to obtain M values, and differentially methylated regions were identified using a p < 0.001 with |*MAT score on T-statistic*| > 2 in ANOVA analysis. Custom scripts were used to produce .wig files which were then visualized in Integrative Genomics Viewer (IGV) for the methylation and expression patterns^116^. Alternatively, differentially expressed and methylated genome regions were also drawn using Partek Genomics Suite.

### Statistical Analysis

Results are presented as Mean ± SD, unless otherwise specified separately in other sections for genomic data analysis. Two‐tailed unpaired Student *t* test was used for comparisons between groups. Differences were considered statistically significant when *p* ≤ 0.05.

### Microarray data

Microarray data can be accessed at the ArrayExpress database with the following accession numbers: E-MTAB-3984, E-MTAB-3982, E-MTAB-3981.

## Additional Information

**How to cite this article**: Zhang, Y. *et al*. Epigenomic Reprogramming of Adult Cardiomyocyte-Derived Cardiac Progenitor Cells. *Sci. Rep*. **5**, 17686; doi: 10.1038/srep17686 (2015).

## Supplementary Material

Supplementary Information

Supplementary Table S1

Supplementary Table S2

Supplementary Table S3

Supplementary Table S4

Supplementary Table S5

Supplementary Table S6

Supplementary Table S7

Supplementary Table S8

Supplementary Table S9

Supplementary Table S10
