# Corrigendum: Epigenomic Reprogramming of Adult Cardiomyocyte-Derived Cardiac Progenitor Cells

**DOI:** 10.1038/srep46907

**Published:** 2017-10-20

**Authors:** Yiqiang Zhang, Jiang F. Zhong, Hongyu Qiu, W. Robb MacLellan, Eduardo Marbán, Charles Wang

Scientific Reports 5: Article number: 17686; 10.1038/srep17686 published online: 12 14 2015; updated: 10 20 2017.

This Article contains errors in the Material and Methods section under the subheading ‘DNA methylation analysis’.

“Genomic DNA was isolated from population adult cardiomyocytes and population mCPCs using Qiagen AllPrep DNA/RNA Micro Kit. Ten (10) ng of genomic DNA was first subject to a whole genome amplification using Sigma’s GenomePlex Complete Whole Genome Amplification (WGA2) kit. The amplified DNA products were used for whole-genome DNA methylome analyses by microarrays using either NimbleGen Mouse 2.1 M Array or NimbleGen Mouse 3 × 720 K CpG Islands Pus RefSeq Promoter Array Tiling Array. Modified standard CHARM protocol with reduced starting amount of amplified genomic DNA (2.5 μg) triplicated in each group was used for two types of arrays^110,111^. Restriction enzyme McrBC was used to digest amplified genomic DNA as it recognizes the site (A/G)^m^C(N_40–3000_)(A/G) ^m^C, with an optimal separation of 55–103 bp, covering nearly half of all possible 5-methylcytosine nucleotides in the genome^112^. For each sample, half of the amplified genomic DNA (2.5 μg) with sizes ranging 100–1000 bp was subjected to McrBC digestion thereby methylated cytosines were cut into smaller fragments. The other half of amplified genomic DNA (2.5 μg) was not treated with McrBC enzyme. Both McrBC-treated and untreated portions were fractionated by 1% agarose gel. The McrBC-treated portion was methyl-depleted (MD) DNA and the untreated (UT) portion represented the total genomic DNA input. Amplified MD (equivalent to experimental sample) and UT (equivalent to input/control sample) samples were labeled with Cy5 and Cy3, respectively, according to standard NimbleGen Array protocol. Labeled DNA samples were hybridized to either types of arrays according to NimbleGen Array User Guide, and the arrays were scanned using MS 200 Microarray Scanner (Roche NimbleGen) and features extracted by NimbleScan software”.

should read:

“Genomic DNA was isolated from population adult cardiomyocytes and population mCPCs using Qiagen AllPrep DNA/RNA Micro Kit. Modified CHARM protocol with reduced starting amount of genomic DNA (500 ng), and amplified gDNA post-McrBC digestion (or without digestion) triplicated in each group, was used for two types of arrays^110,111^. Restriction enzyme McrBC was used to digest genomic DNA as it recognizes the site (A/G)mC(N40–3000)(A/G)mC, with an optimal separation of 55–103 bp, covering nearly half of all possible 5-methylcytosine nucleotides in the genome^112^. For each sample, half of the fragmented gDNA was subjected to McrBC digestion thereby methylated cytosines were cut into smaller fragments; the other half was not treated with McrBC enzyme. Both McrBC-treated and untreated portions were fractionated by 1% agarose gel according to the CHARM protocol. The McrBC-treated portion was methyl-depleted (MD) DNA and the untreated (UT) portion represented the total genomic DNA input. Recovered MD and UT DNA from agarose gel was subsequently amplified using a GenomePlex Complete Whole Genome Amplification (WGA2) kit (Sigma) according to manufacture protocol. Amplified MD (equivalent to experimental sample) and UT (equivalent to input/control sample) samples were labeled with Cy5 and Cy3, respectively, according to standard NimbleGen Array protocol”.

