# Supplementary material for: Epigenomic Reprogramming of Adult Cardiomyocyte-Derived Cardiac Progenitor Cells
Source: Sci Rep. 2015 Dec 14;5:17686. doi: 10.1038/srep17686 (PMC4677315; doi:10.1038/srep17686)
Supplement: Supplementary Information [file srep17686-s1.pdf]

Supplementary Information

Epigenomic Reprogramming of Adult Cardiomyocyte-Derived Cardiac Progenitor Cells

Yiqiang Zhang[1,2§], Jiang F. Zhong[3], Hongyu Qiu[4], W. Robb MacLellan[1], Eduardo Marbán[2], Charles Wang[5*]

[1]Division of Cardiology, Department of Medicine, and Center for Cardiovascular Biology, and Institute for Stem Cell and Regenerative Medicine, University of Washington, Seattle, WA 98109, USA. [2]The Heart Institute, Cedars-Sinai Medical Center, Los Angeles, CA, USA. [3]Department of Pathology, University of Southern California, Los Angeles, CA 90033, USA. [4]Department of Basic Sciences, School of Medicine, Loma Linda University, Loma Linda, CA 92350, USA. [5] Center for Genomics & Department of Basic Sciences, School of Medicine, Loma Linda University, Loma Linda, CA, USA.

[§]Co-corresponding author: YZ (yzhang@cardiology.washington.edu)

*Corresponding author: CW (chwang@llu.edu)

Supplementary Information including:

4 Supplementary Tables, 10 Supplementary Figures, 1 IPA Analysis Report.

Additional information include: 10 Supplementary datasets (MS EXCEL, separated files).

Microarray data can be acceded on the GEO database with accession number GSE49683.

Supplementary Tables

Supplementary Table S1. Cardiac function-, ion channel-, and myofilament structure-related significant genes passed GOEA. Note: For genes with multiple probe sets, the data of maximal Fold Changes (mCPC vs Ctl) are listed.

Gene Symbol	Probeset ID	p-value	Fold-Change	Gene Title	GO Function
Abcc9	1420408_a_at	1.628E-07	-72.5	ATP-binding cassette, sub-family C (CFTR/MRP), member 9	cation channel activity
Acadm	1415984_at	8.057E-05	-52.5	acyl-Coenzyme A dehydrogenase, medium chain	cardiac muscle cell differentiation
Actc1	1415927_at	8.242E-06	-112.4	actin, alpha, cardiac muscle 1	cardiac muscle contraction
Actn2	1448327_at	3.007E-05	-34.8	actinin alpha 2	ion channel binding
Adk	1416319_at	3.867E-04	-11.5	adenosine kinase	positive regulation of cardiac muscle hypertrophy
Akap6	1440859_at	2.943E-04	-15.4	A kinase (PRKA) anchor protein 6	regulation of cardiac muscle cell contraction
Ank2	1434265_s_at	7.025E-04	-13.9	ankyrin 2, brain	regulation of heart rate by cardiac conduction
Asph	1425274_at	5.640E-04	-7.5	aspartate-beta-hydroxylase	positive regulation of cation channel activity
Atp1a2	1443823_s_at	4.994E-06	-9.4	ATPase, Na+/K+ transporting, alpha 2 polypeptide	regulation of cardiac muscle cell contraction
Cacna1c	1421297_a_at	4.424E-05	-16.5	calcium channel, voltage-dependent, L type, alpha 1C subunit	cation channel activity
Cacna2d1	1449999_a_at	1.053E-04	-10.1	calcium channel, voltage-dependent, alpha2/delta subunit 1	cation channel activity

Cacnb2	1456401_at	2.424E-04	-3.9	calcium channel, voltage-dependent, beta 2 subunit	cation channel activity
Casq2	1422529_s_at	4.512E-05	-51.2	calsequestrin 2	regulation of cardiac muscle cell contraction
Clic5	1456873_at	2.073E-05	-34.6	chloride intracellular channel 5	voltage-gated anion channel activity
Csrp3	1460318_at	1.464E-05	-36.8	cysteine and glycine-rich protein 3	cardiac muscle contraction
Dmd	1417307_at	7.182E-04	-20.5	dystrophin, muscular dystrophy	regulation of cation channel activity
Dsc2	1426911_at	8.223E-05	-21.4	desmocollin 2	regulation of heart rate by cardiac conduction
Dsg2	1426153_a_at	2.562E-04	-20.5	desmoglein 2	regulation of heart rate by cardiac conduction
Dsp	1435494_s_at	8.405E-07	-172.1	desmoplakin	regulation of heart rate by cardiac conduction
Fgf1	1450869_at	8.131E-04	-9.2	fibroblast growth factor 1	cardiac muscle cell proliferation
Fgf13	1418497_at	1.235E-04	-12.7	fibroblast growth factor 13	ion channel binding
Fhod3	1435551_at	8.651E-05	-17.0	formin homology 2 domain containing 3	cardiac myofibril assembly
Hsp90aa1	1437497_a_at	5.360E-04	5.8	heat shock protein 90, alpha (cytosolic), class A member 1	ion channel binding
Kcnj3	1455374_at	1.671E-04	-18.0	potassium inwardly-rectifying channel, subfamily J, member 3	cation channel activity
Kcnj5	1441590_at	9.423E-05	-18.1	potassium inwardly-rectifying channel, subfamily J, member 5	cation channel activity
Mef2c	1421027_a_at	3.611E-04	-19.9	myocyte enhancer factor 2C	positive regulation of cardiac muscle cell proliferation
Murc	1420551_at	7.891E-05	-9.8	muscle-related coiled-coil protein	cardiac myofibril assembly

Mybpc3	1418551_at	2.182E-05	-20.6	myosin binding protein C, cardiac	cardiac muscle contraction
Myh6	1448826_at	2.863E-05	-170.2	myosin, heavy polypeptide 6, cardiac muscle, alpha	cardiac muscle cell development
Myh7	1448553_at	6.507E-05	-10.0	myosin, heavy polypeptide 7, cardiac muscle, beta	ventricular cardiac muscle tissue morphogenesis
Myl2	1448394_at	3.963E-05	-107.1	myosin, light polypeptide 2, regulatory, cardiac, slow	cardiac muscle contraction
Myl3	1427768_s_at	9.240E-05	-29.3	myosin, light polypeptide 3	cardiac muscle contraction
Mylk3	1439101_at	1.033E-04	-20.1	myosin light chain kinase 3	cardiac myofibril assembly
Myocd	1425978_at	2.946E-04	-15.0	myocardin	cell growth involved in cardiac muscle cell development
Nexn	1435649_at	2.471E-04	-35.3	nexilin	cardiac muscle cell development
Phpt1	1427903_at	2.574E-04	-6.7	phosphohistidine phosphatase 1	ion channel inhibitor activity
Pln	1460332_at	1.636E-05	-171.5	phospholamban	regulation of the force of heart contraction by cardiac conduction
Popdc2	1417806_at	7.431E-04	-14.1	popeye domain containing 2	cardiac pacemaker cell development
Prox1	1437894_at	8.123E-04	-10.1	prospero-related homeobox 1	cardiac septum morphogenesis
Rgs2	1419248_at	6.096E-05	-22.9	regulator of G-protein signaling 2	positive regulation of cardiac muscle contraction
Ryr2	1450123_at	3.973E-05	-106.9	ryanodine receptor 2, cardiac	positive regulation of ryanodine-sensitive calcium-release channel activity
Sgcb	1419668_at	1.464E-05	-12.0	sarcoglycan, beta (dystrophin-associated	cardiac muscle cell development

				glycoprotein)	
Sox4	1455867_at	2.081E-04	3.8	SRY-box containing gene 4	cardiac septum morphogenesis
Tgfbr3	1433795_at	1.208E-04	-5.0	transforming growth factor, beta receptor III	cardiac epithelial to mesenchymal transition
Tnnc1	1418370_at	4.610E-05	-20.0	troponin C, cardiac/slow skeletal	cardiac muscle contraction
Tnni3	1422536_at	2.789E-05	-130.7	troponin I, cardiac 3	cardiac muscle contraction
Tnnt2	1424967_x_at	1.741E-04	-85.1	troponin T2, cardiac	myofilament
Trdn	1451801_at	1.283E-06	-37.8	triadin	cation channel complex
Ttn	1444083_at	6.915E-07	-74.2	titin	cardiac muscle contraction

Supplementary Table S2. Cell cycle-related significant genes passed GOEA. Note: For genes with multiple probe sets, the data of maximal Fold Changes (mCPC vs Ctl) are listed.

Gene Symbol	Probeset ID	p-value	Fold-Change	Gene Title	GO Function
Ank3	1451628_a_at	4.57E-05	-5.6	ankyrin 3, epithelial	cell cycle process
Anxa1	1448213_at	2.86E-05	47.7	annexin A1	positive regulation of cell cycle process
Appl2	1426743_at	3.12E-05	-6.4	adaptor protein, phosphotyrosine interaction, PH domain and leucine zipper containing 2	cell cycle
Ccnd2	1430127_a_at	0.001452	5.5	cyclin D2	cell cycle
Ccnl2	1432195_s_at	0.000576	-2.2	cyclin L2	regulation of cell cycle
Cdk4	1422439_a_at	0.039159	8.0	cyclin-dependent kinase 4	regulation of cell cycle
Cdkn1a	1424638_at	3.29E-06	28.6	cyclin-dependent kinase inhibitor 1A (P21)	positive regulation of cell cycle process
Cdkn1b	1434045_at	0.000706	-3.0	cyclin-dependent kinase inhibitor 1B	G1/S transition of mitotic cell cycle
Cfl1	1455138_x_at	0.000323	7.9	cofilin 1, non-muscle	cell cycle process
Cks2	1417457_at	0.000414	47.8	CDC28 protein kinase regulatory subunit 2	cell cycle
Clasp2	1456911_at	0.000379	-2.7	CLIP associating protein 2	cell cycle
Csnk1d	1418889_a_at	0.000543	6.8	casein kinase 1, delta	cell cycle process
Cul3	1434717_at	0.000267	-5.3	cullin 3	positive regulation of cell cycle process
Ddit3	1443897_at	0.000258	-14.0	DNA-damage inducible transcript 3	cell cycle arrest
Ereg	1419431_at	4.90E-05	37.7	epiregulin	positive regulation of cell cycle process
Esr1	1421244_at	0.000176	-75.3	estrogen receptor 1 (alpha)	regulation of mitotic cell cycle
Fbxw7	1451558_at	0.000139	-8.9	F-box and WD-40 domain protein 7	regulation of cell cycle
Fosl1	1417487_at	1.84E-05	39.4	fos-like antigen 1	regulation of cell cycle
Gadd45b	1450971_at	0.000319	21.3	growth arrest and DNA-damage-inducible 45 beta	regulation of cell cycle
Hdac3	1426437_s_at	8.62E-05	-2.4	histone deacetylase 3	regulation of mitotic cell cycle
Insr	1450225_at	9.74E-05	-8.0	insulin receptor	positive regulation of cell cycle process
Lats2	1419678_at	0.000769	3.4	large tumor suppressor 2	G1/S transition of mitotic cell cycle
Mlf1	1418589_a_at	4.19E-05	-18.2	myeloid leukemia factor 1	cell cycle arrest
Mtus1	1454824_s_at	3.27E-06	-25.8	mitochondrial tumor suppressor 1	cell cycle

Myc	1424942_a_at	5.99E-07	32.3	myelocytomatosis oncogene	regulation of mitotic cell cycle
Myocd	1425978_at	0.000295	-15.0	myocardin	regulation of cell cycle
Ncor1	1423200_at	0.000654	-3.8	nuclear receptor co-repressor 1	cell cycle process
Npm1	1415839_a_at	0.000259	3.8	nucleophosmin 1	positive regulation of cell cycle process
Nsun2	1423850_at	0.000409	5.5	NOL1/NOP2/Sun domain family member 2	cell cycle
Pbx1	1428647_at	0.000738	-6.4	pre B-cell leukemia transcription factor 1	positive regulation of cell cycle process
Pdpn	1419309_at	0.000755	23.5	podoplanin	regulation of mitotic cell cycle
Prox1	1437894_at	0.000812	-10.1	prospero-related homeobox 1	positive regulation of cell cycle process
Rb1cc1	1418968_at	7.59E-05	-10.6	RB1-inducible coiled-coil 1	cell cycle
Rgs2	1419248_at	6.10E-05	-22.9	regulator of G-protein signaling 2	cell cycle
Sox4	1455867_at	0.000208	3.8	SRY-box containing gene 4	positive regulation of cell cycle process
Spast	1460400_at	6.44E-05	-5.1	spastin	cell cycle
Sphk1	1451596_a_at	0.000519	4.5	sphingosine kinase 1	regulation of mitotic cell cycle
Tacc2	1425745_a_at	1.98E-05	-6.2	transforming, acidic coiled-coil containing protein 2	cell cycle process
Taok1	1455432_at	0.0006	-8.3	TAO kinase 1	cell cycle checkpoint
Thbs1	1421811_at	0.000438	74.8	thrombospondin 1	cell cycle arrest
Tubb5	1416256_a_at	0.000537	15.7	tubulin, beta 5	cell cycle process
Txnip	1415996_at	0.000187	-5.2	thioredoxin interacting protein	cell cycle
Usp9x	1450038_s_at	0.000153	-19.9	ubiquitin specific peptidase 9, X chromosome	cell cycle

Supplementary Table S3. Cell proliferation-related genes passed GOEA. For genes with multiple probe sets, the data of maximal Fold Changes (mCPC vs Ctl) are listed. All genes are in "cell proliferation" on GO function catalogue.

Gene Symbol	Probeset ID	p-value	Fold-Change	Gene Title	GO Function
Adk	1416319_at	0.00039	-11.4	adenosine kinase	positive regulation of cell proliferation
Anxa1	1448213_at	2.86E-05	47.7	annexin A1	regulation of cell proliferation
Appl2	1426743_at	3.12E-05	-6.4	adaptor protein, phosphotyrosine interaction, PH domain and leucine zipper containing 2	cell proliferation
Asph	1425274_at	0.00056	-7.5	aspartate-beta-hydroxylase	negative regulation of cell proliferation
Bche	1421218_at	0.00039	-9.5	butyrylcholinesterase	negative regulation of cell proliferation
Cd81	1416330_at	0.0002	-2.8	CD81 antigen	positive regulation of cell proliferation
Cdkn1a	1424638_at	3.29E-06	28.6	cyclin-dependent kinase inhibitor 1A (P21)	negative regulation of cell proliferation
Cdkn1b	1434045_at	0.00071	-3.0	cyclin-dependent kinase inhibitor 1B	negative regulation of cell proliferation
Csf1	1460220_a_at	0.00035	16.8	colony stimulating factor 1 (macrophage)	positive regulation of cell proliferation
Dfna5	1421534_at	0.0002	-7.3	deafness, autosomal dominant 5 (human)	negative regulation of cell proliferation
Entpd5	1417382_at	1.28E-06	-41.8	ectonucleoside triphosphate diphosphohydrolase 5	cell proliferation
Ereg	1419431_at	4.90E-05	37.7	epiregulin	negative regulation of cell proliferation
Esr1	1421244_at	0.00018	-75.3	estrogen receptor 1 (alpha)	positive regulation of cell proliferation
Fabp4	1451263_a_at	0.00084	-30.6	fatty acid binding protein 4, adipocyte	positive regulation of cell proliferation
Fgf1	1450869_at	0.00081	-9.2	fibroblast growth factor 1	positive regulation of cell proliferation
Fgf13	1418497_at	0.00012	-12.7	fibroblast growth factor 13	cell proliferation
Fosl1	1417487_at	1.84E-05	39.4	fos-like antigen 1	negative regulation of cell proliferation
Foxo3	1434832_at	0.00014	-9.3	forkhead box O3	regulation of cell proliferation
Gm13654 /// Gm16409 /// LOC639593 /// Rps6	1437246_x_at	0.00055	2.6	predicted gene 13654 /// ribosomal protein S6 pseudogene /// 40S ribosomal protein S6-l	cell proliferation
Gpam	1419499_at	5.46E-05	-28.4	glycerol-3-phosphate acyltransferase, mitochondrial	positive regulation of cell proliferation
Hipk2	1428433_at	2.38E-06	-66.3	homeodomain interacting protein kinase 2	positive regulation of cell proliferation
Hmox1	1448239_at	0.00074	23.3	heme oxygenase (decycling) 1	negative regulation of cell proliferation

Igfbp5	1422313_a_at	0.00014	-7.0	insulin-like growth factor binding protein 5	negative regulation of cell proliferation
Insr	1450225_at	9.74E-05	-8.0	insulin receptor	positive regulation of cell proliferation
Mef2c	1421027_a_at	0.00036	-19.9	myocyte enhancer factor 2C	negative regulation of cell proliferation
Mitf	1455214_at	0.00018	-8.9	microphthalmia-associated transcription factor	regulation of cell proliferation
Mtss1	1434036_at	0.00048	-3.5	metastasis suppressor 1	negative regulation of cell proliferation
Myc	1424942_a_at	5.99E-07	32.3	myelocytomatosis oncogene	positive regulation of metanephric cap mesenchymal cell proliferation
Myocd	1425978_at	0.00029	-15.0	myocardin	negative regulation of cell proliferation
Nampt	1448607_at	7.39E-06	-20.3	nicotinamide phosphoribosyltransferase	positive regulation of cell proliferation
Ncor1	1423200_at	0.00065	-3.8	nuclear receptor co-repressor 1	positive regulation of cell proliferation
Ndrg2	1448154_at	0.00034	-51.0	N-myc downstream regulated gene 2	negative regulation of cell proliferation
Ndufaf4	1435333_at	0.00039	-10.4	NADH dehydrogenase (ubiquinone) 1 alpha subcomplex, assembly factor 4	positive regulation of cell proliferation
Npm1	1415839_a_at	0.00026	3.8	nucleophosmin 1	negative regulation of cell proliferation
Pbx1	1428647_at	0.00074	-6.4	pre B-cell leukemia transcription factor 1	positive regulation of cell proliferation
Pdk1	1423747_a_at	0.00016	-31.8	pyruvate dehydrogenase kinase, isoenzyme 1	cell proliferation
Pdpn	1419309_at	0.00076	23.5	podoplanin	cell proliferation
Pik3r1	1425515_at	8.26E-05	-15.4	phosphatidylinositol 3-kinase, regulatory subunit, polypeptide 1 (p85 alpha)	negative regulation of cell proliferation
Pmaip1	1418203_at	0.00032	49.0	phorbol-12-myristate-13-acetate-induced protein 1	negative regulation of cell proliferation
Ppargc1a	1434099_at	0.0006	-22.6	peroxisome proliferative activated receptor, gamma, coactivator 1 alpha	positive regulation of cell proliferation
Prox1	1437894_at	0.00081	-10.1	prospero-related homeobox 1	negative regulation of cell proliferation
Rnf139	1429425_at	0.00024	-22.2	ring finger protein 139	negative regulation of cell proliferation
Rora	1436325_at	7.41E-05	-3.1	RAR-related orphan receptor alpha	cell proliferation
Serpine1	1419149_at	0.00072	11.3	serine (or cysteine) peptidase inhibitor, clade E, member 1	positive regulation of cell proliferation
Sod2	1417194_at	1.31E-05	-5.5	superoxide dismutase 2, mitochondrial	negative regulation of cell proliferation
Sox4	1455867_at	0.00021	3.8	SRY-box containing gene 4	negative regulation of cell proliferation
Sphk1	1451596_a_at	0.00052	4.5	sphingosine kinase 1	positive regulation of cell proliferation
Suz12	1452364_at	0.00058	-2.7	suppressor of zeste 12 homolog (Drosophila)	positive regulation of cell proliferation

Tacc2	1425745_a_at	1.98E-05	-6.2	transforming, acidic coiled-coil containing protein 2	cell proliferation
Tgfb2	1450922_a_at	0.01348	6.0	transforming growth factor, beta 2	cell proliferation
Tgfbr3	1433795_at	0.00012	-5.0	transforming growth factor, beta receptor III	negative regulation of cell proliferation
Thbs1	1421811_at	0.00044	74.8	thrombospondin 1	negative regulation of cell proliferation
Tial1	1452821_at	6.03E-05	-6.1	Tia1 cytotoxic granule-associated RNA binding protein-like 1	positive regulation of cell proliferation
Txnip	1415996_at	0.00019	-5.2	thioredoxin interacting protein	regulation of cell proliferation
Yme1l1	1450954_at	0.00071	-7.2	YME1-like 1 (S. cerevisiae)	cell proliferation
Zeb1	1418926_at	0.00074	-9.1	zinc finger E-box binding homeobox 1	negative regulation of cell proliferation

Supplementary Table S4. Stem cell related genes passed GOEA. Note: For genes with multiple probe sets, the data of maximal Fold Changes (mCPC vs Ctl) are listed.

Gene Symbol	Probeset ID	p-value	Fold-Change	Gene Title	GO Function
Bche	1421218_at	0.00039	-9.5	butyrylcholinesterase	stem cell differentiation
Col1a1	1423669_at	2.67E-05	10.1	collagen, type I, alpha 1	regulation of stem cell differentiation
Cul3	1434717_at	0.000267	-5.3	cullin 3	stem cell division
Hook3	1435157_at	0.000148	-4.7	hook homolog 3 (Drosophila)	neuronal stem cell maintenance
Mef2c	1421027_a_at	0.000361	-19.9	myocyte enhancer factor 2C	stem cell differentiation
Msi2	1435520_at	1.78E-05	-21.8	Musashi homolog 2 (Drosophila)	stem cell development
Mtf2	1418515_at	0.00017	-3.2	metal response element binding transcription factor 2	stem cell differentiation
Myc	1424942_a_at	5.99E-07	32.3	myelocytomatosis oncogene	regulation of stem cell proliferation
Pcm1	1449120_a_at	0.000723	-2.8	pericentriolar material 1	neuronal stem cell maintenance
Prox1	1437894_at	0.000812	-10.1	prospero-related homeobox 1	neuronal stem cell maintenance
Sox4	1455867_at	0.000208	3.8	SRY-box containing gene 4	somatic stem cell maintenance
Tgfb2	1450922_a_at	0.0134806	6.0	Transforming growth factor, beta 2	Somatic stem cell division
Tgfbr3	1433795_at	0.000121	-5.0	transforming growth factor, beta receptor III	regulation of stem cell differentiation
Tial1	1452821_at	6.03E-05	-6.1	Tia1 cytotoxic granule-associated RNA binding protein-like 1	stem cell division
Zeb1	1418926_at	0.000742	-9.1	zinc finger E-box binding homeobox 1	regulation of stem cell proliferation

Supplementary Figures

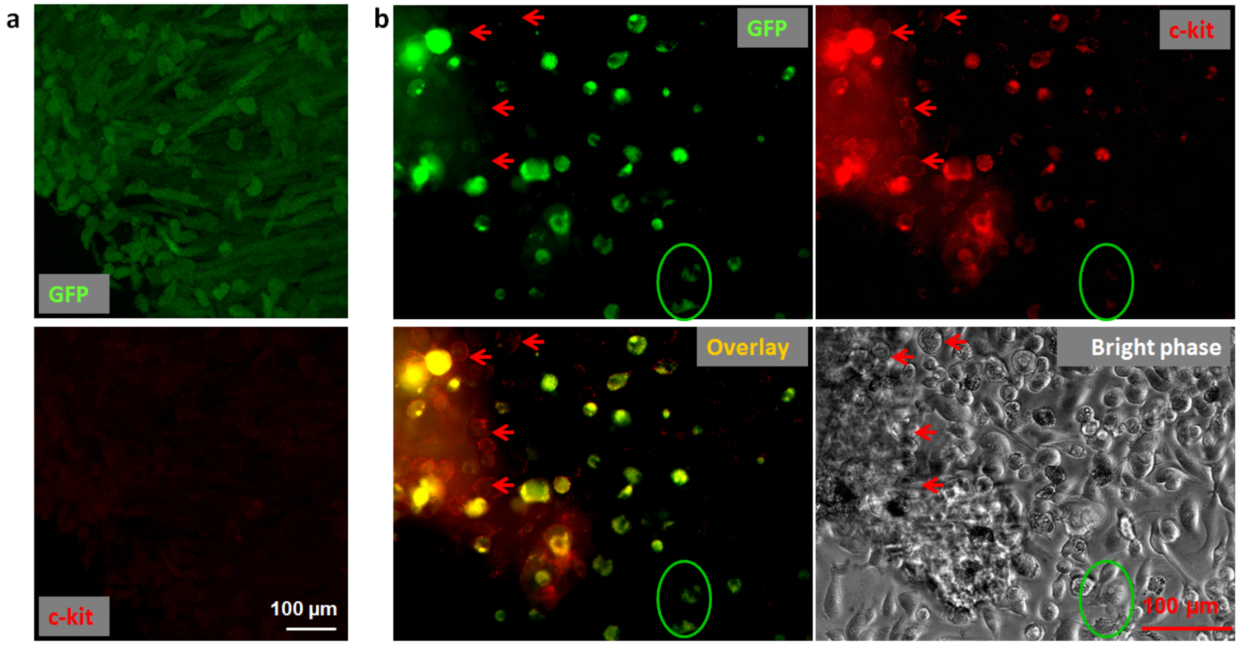

Supplementary Figure S1. Fluorescent immunocytochemistry of cardiac explant culture. (**a**) Cardiac explant culture at the first day of plating, showing GFP+ cardiomyocytes in rode shape and some started to round up, with no visible c-kit signal when detected by antibody. (**b**) Cardiac explant culture at 2 weeks, showing the coexpression of GFP (green) and c-kit (red) in myocyte-derived progenitor cells. Red arrows indicates the resident c-kit$^+$ cells that do not expressing GFP, and the green circle indicates the GFP$^+$-myocyte progeny that have no or very low expression of c-kit. Scale bars= 100 µm.

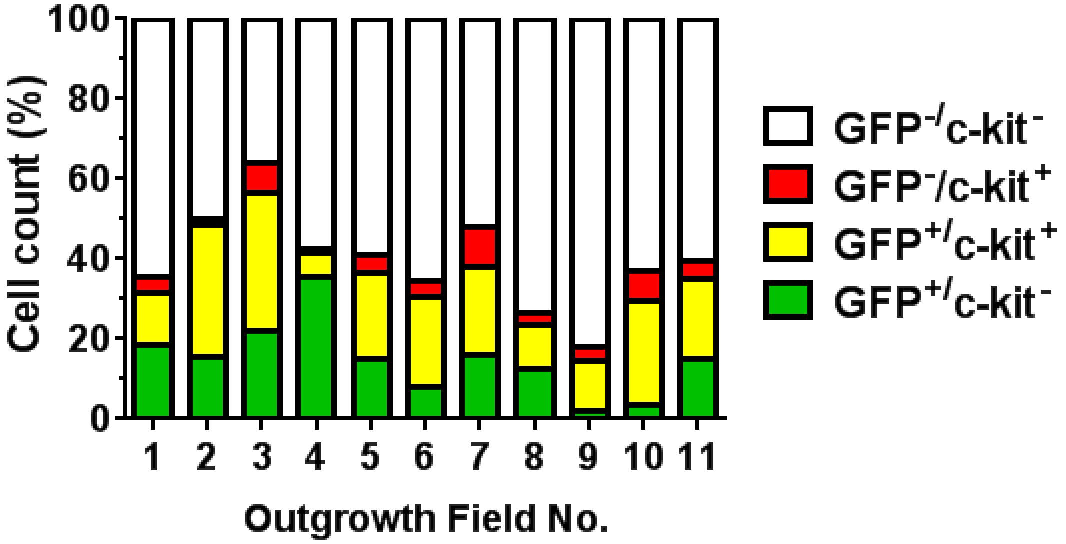

Supplementary Figure S2. The expression of GFP and c-kit in outgrowth of cardiac explant. Shown is the percentage of the cells without or with GFP and/or c-kit expression in measured remote fields 2 weeks after cardiac explant culture (N=2 hearts). There were 60.3%±3.7% cells in GFP^-/c-kit^-, 4.5%±2.6% in GFP^-/c-kit^+, 20.3%±9.0% in GFP^+/c-kit^+, and 14.8%±9.2% in GFP^+/c-kit^-.

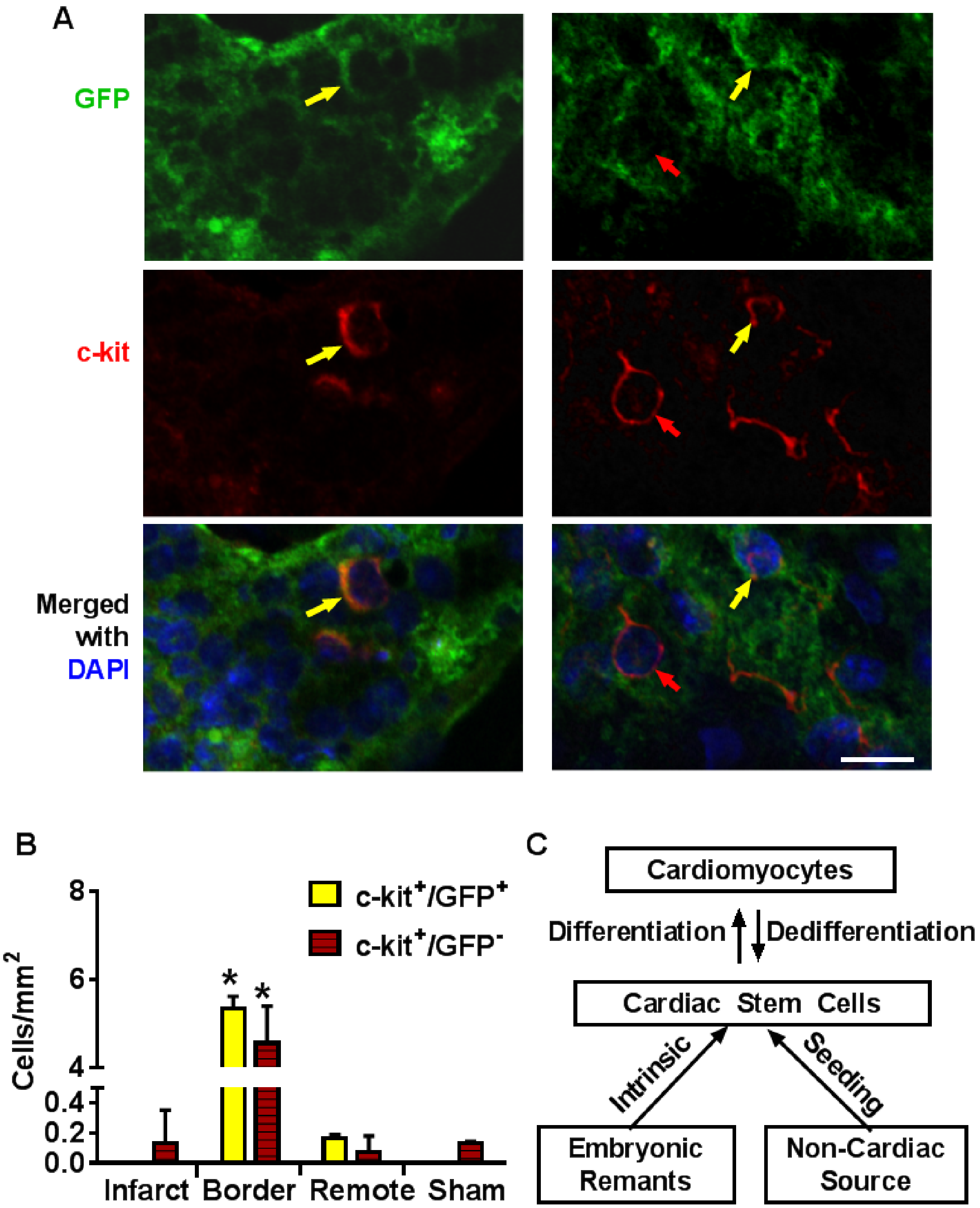

Supplementary Figure S3. Dedifferentiation of cardiomyocytes *in vivo*. Adult MerCreMer-Z/EG bitransgenic mice treated with 4-OH-tamoxifen were subjected to LAD ligation to create infarction. (**a**) Immunohistochemistry showing the c-kit^{+} (red) cells with (yellow arrow) or without (red arrows) the expression of GFP (green) in the infarct border zone. Scale bar, 10 µm. (**b**) Regional difference in the frequency of cells expressing c-kit and/or GFP. *$p < 0.05$ vs.

infarct or remote area. (**c**) Paradigms for cardiac regeneration. Cardiomyocytes and other heart cells can be developed and differentiated from cardiac progenitor/stem cells of either intrinsic embryonic remnants or those seeded from non-cardiac sources. Heart cells can also dedifferentiate and become plastic to contribute to the cardiac progenitor cell pools, such that the cellular dynamics is maintained.

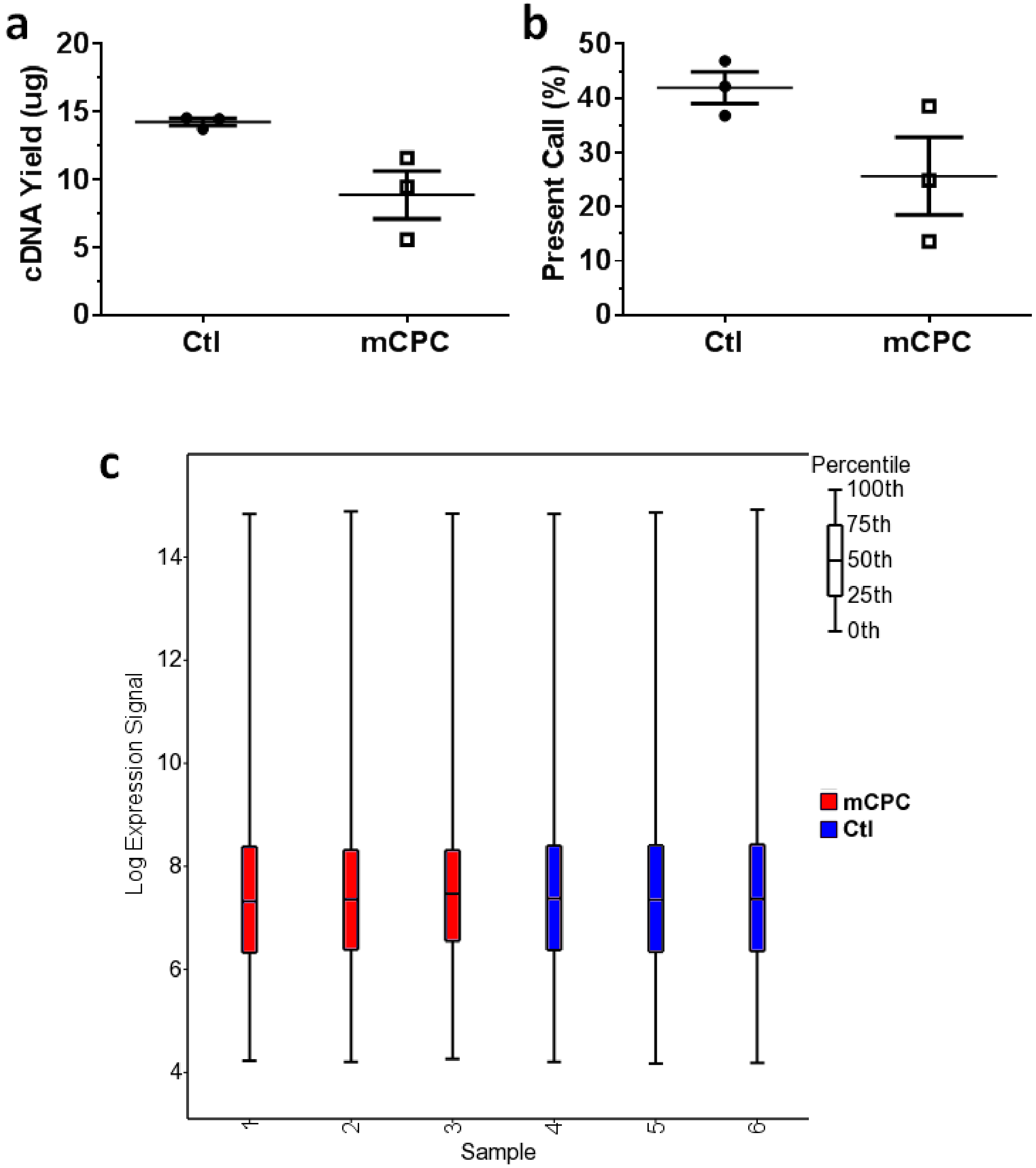

Supplementary Figure S4. Quality control on single-cell microarray processes. (a) cDNA yields from single cardiomyocytes (Ctl) and myocytes-derived progenitor cells (mCPC). **(b)** Present call of probe sets in the microarray. N=3 single cells for each group. (**c**) Box plot on Log expression signal.

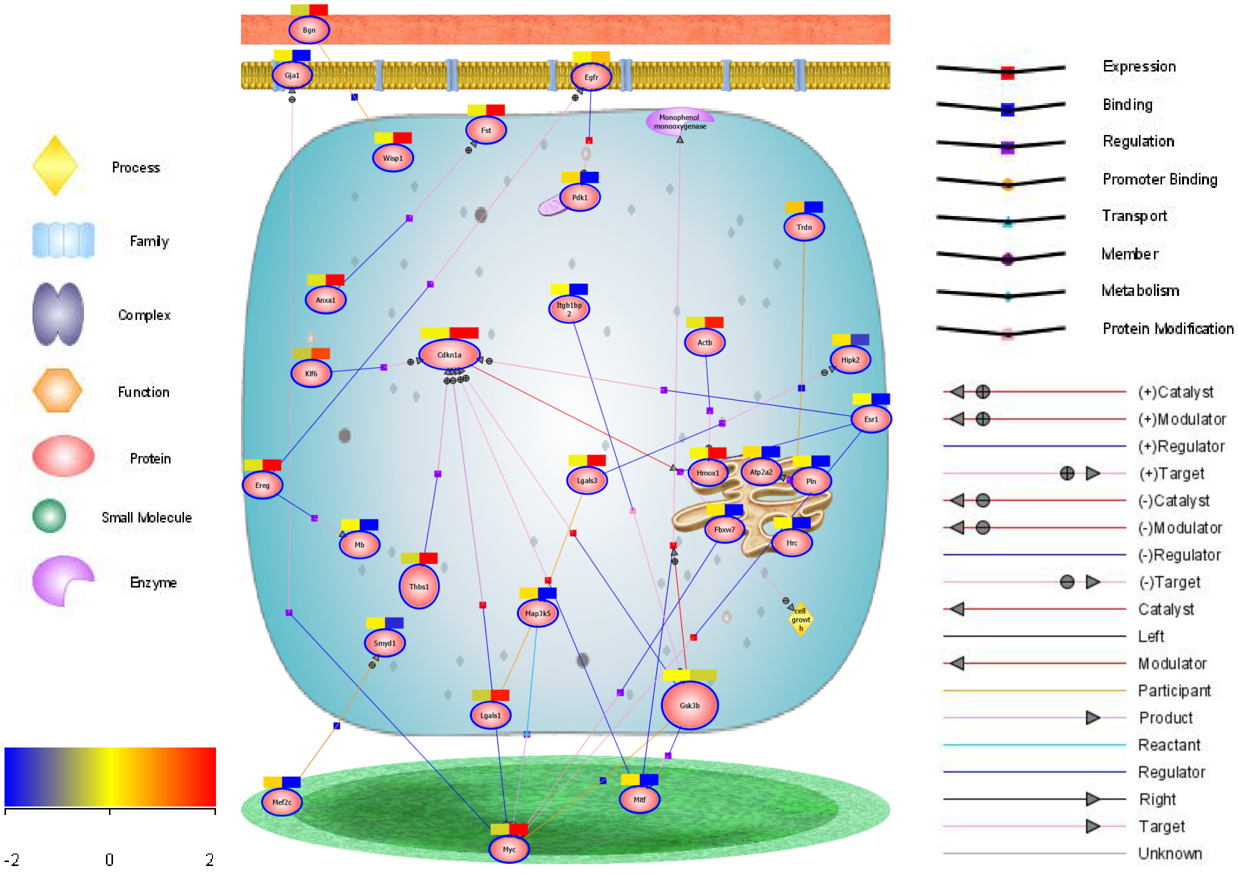

Supplemental Figure S5. Pathway analysis based on differentially expressed genes (DEGs). Significant genes (DEGs) were applied into Pathway Analysis module using Genespring GX 11 (Agilent). Algorithm of Direct interactions on all relation types with a relation score ≥ 9 was included for analysis. As shown in the cellular view, among the 41 relations, there were 3 hubs Gsk3b, Cdnk1a (p21), and Myc which played central roles in cell fate change of cardiomyocyte (Ctl) into myocyte-derived cardiac progenitors cells (mCPCs). Color bars over the genes indicate the expression levels in Ctl (left bar) and mCPCs (right bar).

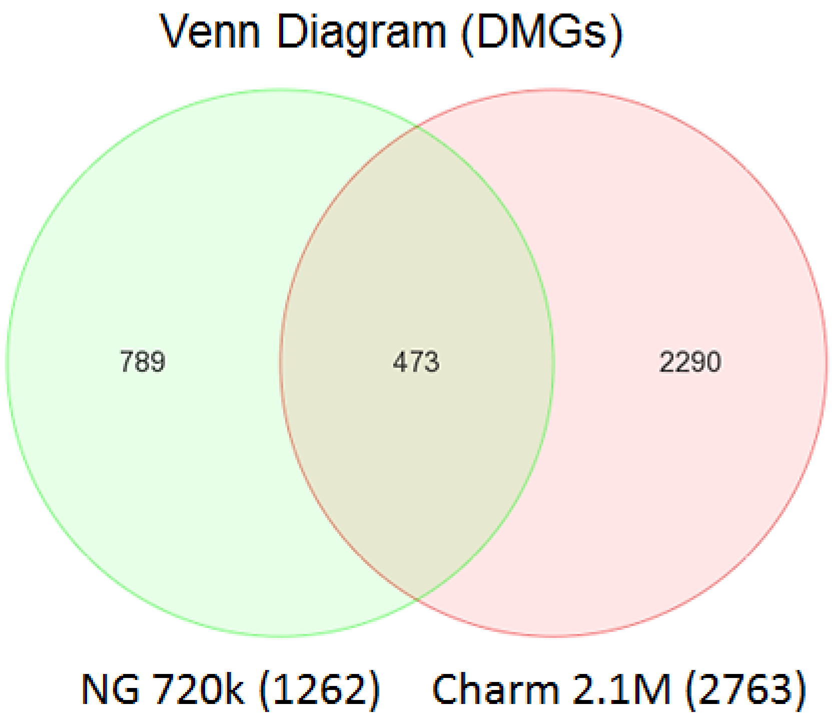

Supplemental Figure S6. Venn diagram of the differentially-methylated genes (DMGs) corresponded to differentially-methylated regions when comparing mCPCs with adult myocyte. DNA methylation was detected by the NimbleGen 720k array and the CHARM customer design 2.1M array.

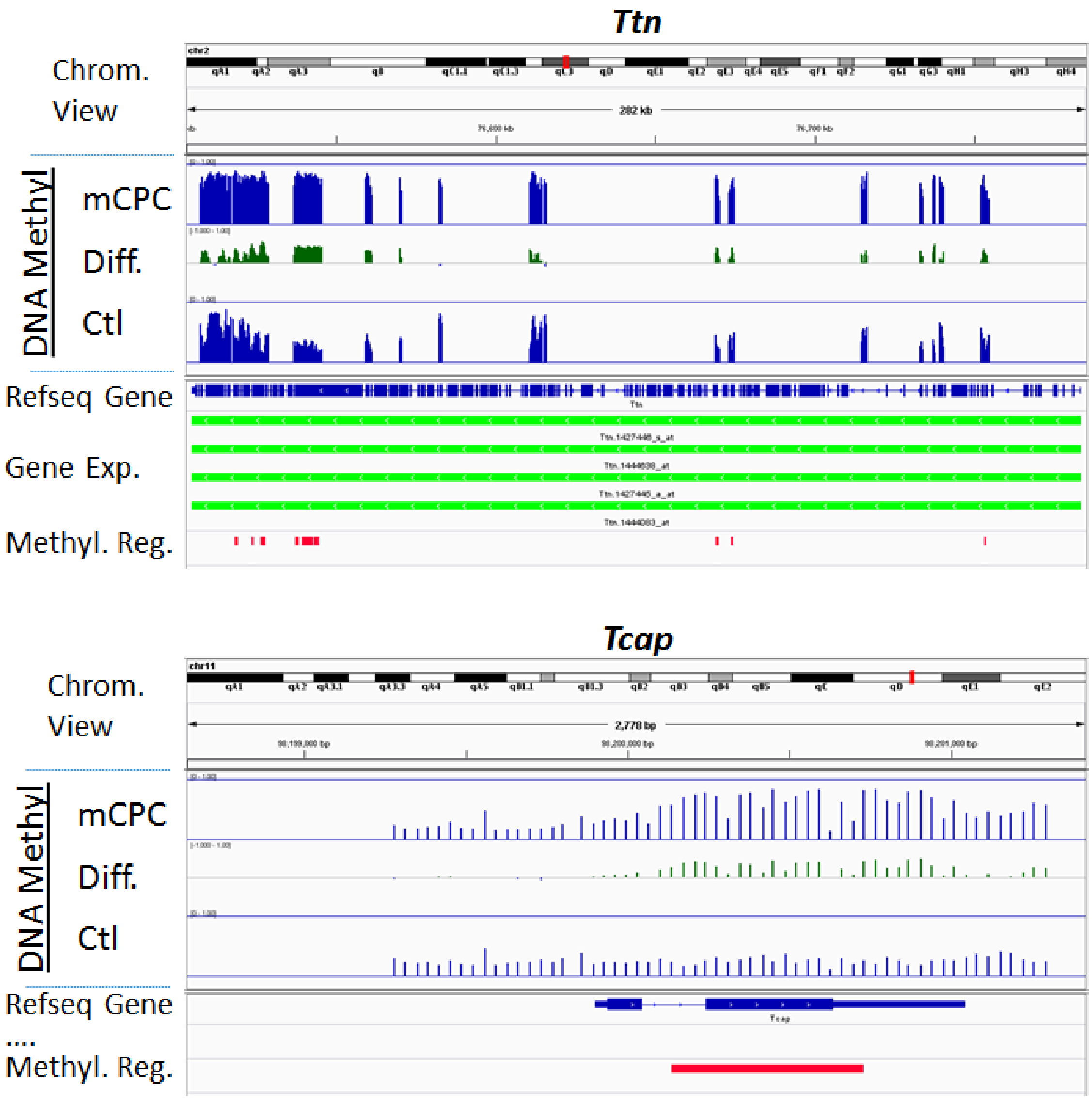

Supplementary Figure S7: Potential epigenetic regulation of Titin (*ttn*) and Titin-cap (*Tcap*). DNA methylation and gene expression array data is visualized with IGV on Titin (*Ttn*, top panel) and Titin-cap gene (*Tcap*, bottom panel) showing the hypermethylation of the *Ttn* and *Tcap* gene body. For both panels, top portion shows the chromosomal region flanking *Tcap* gene. The middle bar graphs present the DNA methylation levels (scale 0-1.0) assayed by CHARM customer array in mCPCs and control myocytes as well the differential methylation level (Diff.) comparing mCPC to Ctl. The bottom portion shows the Refseq gene structures and the differentially-methylated region in mCPCs vs myocytes. *Tcap* gene was down-regulated (-6.6 fold) in mCPCs compared to control (Ctl) myocytes, with p=0.054098 so not shown in the transcription level (fold-change) section. Gene expression array data has been converted to match same genome built (mm8) as CHARM array.

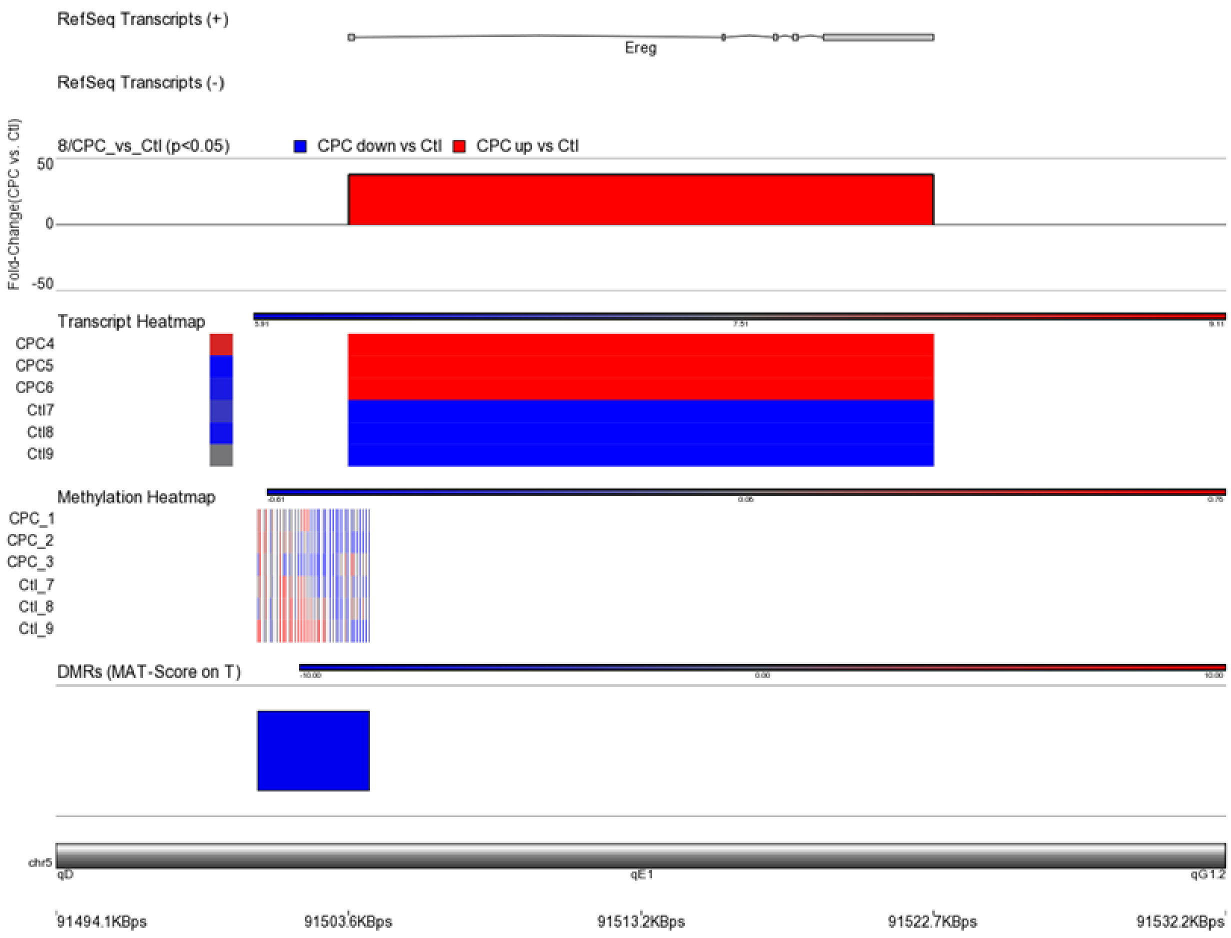

Supplementary Figure S8: Potential epigenetic regulation of Epiregulin (*Ereg*). Genomic view of *Ereg* which is up-regulated in CPCs compared to control myocytes but the promoter regions being methylated. The top line shows the gene structure and *ereg* is on the plus strand. The 2nd and 3rd lines: fold changes and transcript heatmap of CPCs vs myocytes detected by Affymetrix Genechip gene expression array. The 4th and 5th lines: DNA methylation heatmap, and DMR based on MAT-score on T detected by Roche 3x720k DNA methylation array. The bottom panel: genome location.

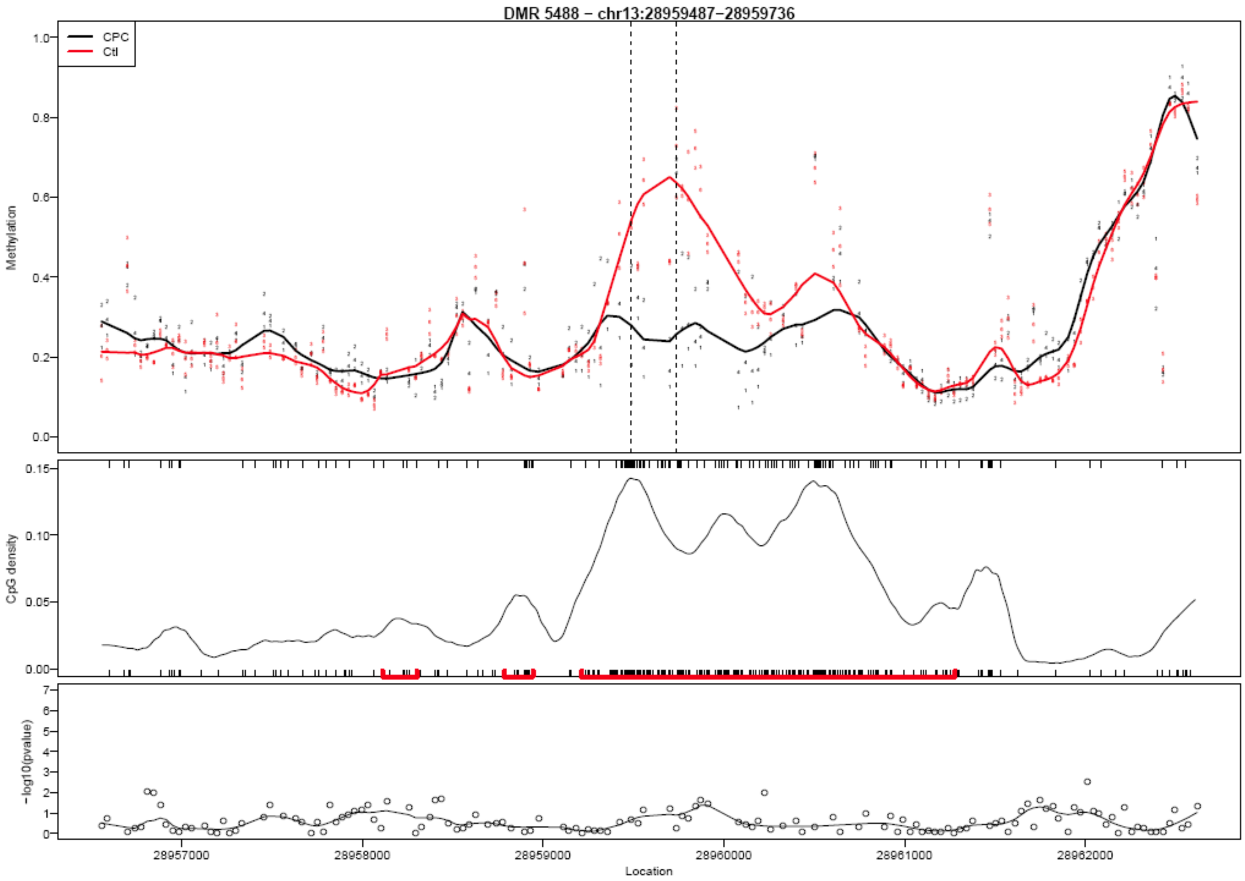

Supplementary Figure S9. Epigenetic Regulation of *Sox4*. The upper panel shows the methylation level (1.0=100%) of adult cardiomyocytes (Ctl, red) and their derived cardiac progenitor cells (mCPCs, black). The vertical dash lines define the edges of the DMR. The middle panel shows the CpG density; with the red brackets represent the CpG islands. Lower panel shows the chromosome locations and differential methylation p values for probes.

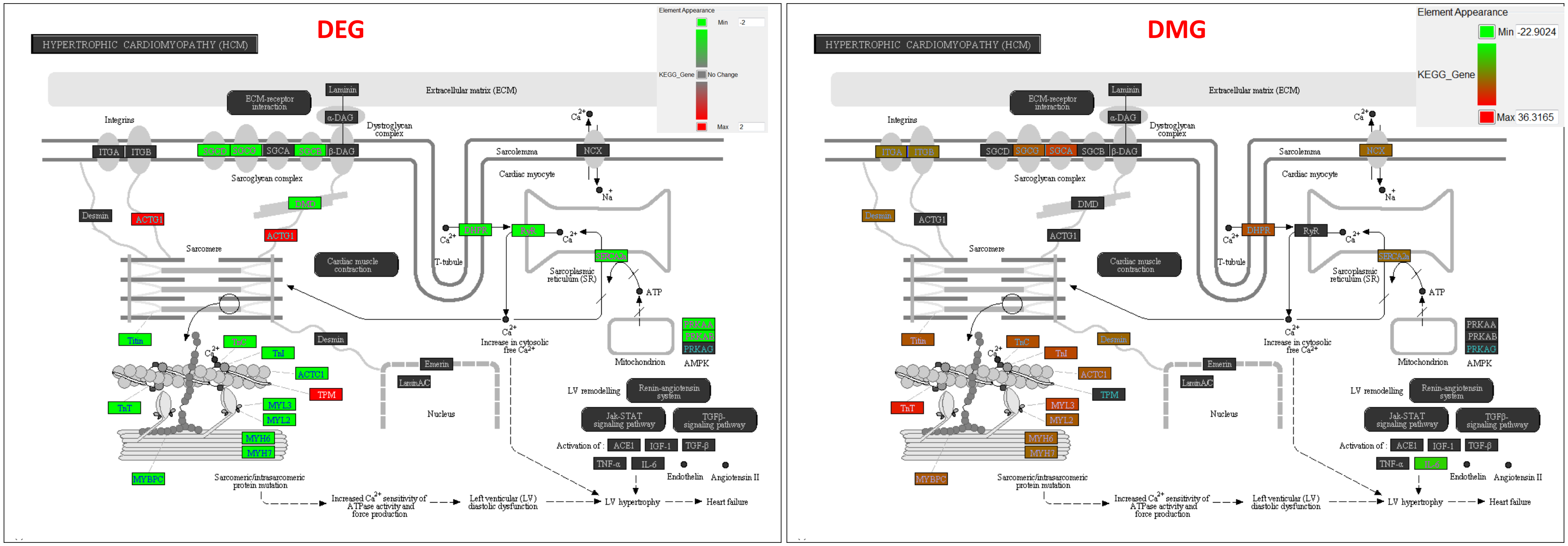

Supplementary Figure S10. Integrated pathway enrichment analysis based on differentially expressed genes (DEGs) and differentially methylated genes (DMGs). The "cardiac hypertrophy pathway" is enriched by the DEGs and DMGs based on KEGG knowledge database using the DEGs detected by Affymetrix GeneChIP and the DMGs detected by NimbleGen 720k DNA methylation array ($p<0.05$, |MAT Score-on-T statics|>10). The down-regulation of most genes in this pathway (left: green color shaded genes) in mCPCs vs adult myocytes is well orchestrated with their DNA hypermethylation (right; red to brown color shaded genes). The enrichment p value for hypertrophic cardiomyopathy pathway is 2.2×10^{-6} for gene expression and 7.6×10^{-7} for DNA methylation.

Analysis Name: MDC vs. Ctl FC2FDR05.txt [Passed]
Analysis Creation Date: 2014-09-26
Build version: 313398M
Content version: 18841524 (Release Date: 2014-06-24)

Analysis settings

View

Reference set: Ingenuity Knowledge Base (Genes Only)

Relationship to include: Direct and Indirect

Does not Include Endogenous Chemicals

Optional Analyses: My Pathways My List

Filter Summary:

Consider only relationships where

confidence = Experimentally Observed

Cutoff:

(c) 2000-2014 QIAGEN. All rights reserved.

Top Canonical Pathways

Name	p-value	Ratio
Calcium Signaling	9.58E-07	18/178 (0.101)
Mitochondrial Dysfunction	1.04E-05	16/171 (0.094)
Epithelial Adherens Junction Signaling	2.8E-05	14/146 (0.096)
Aryl Hydrocarbon Receptor Signaling	7.5E-05	13/140 (0.093)
Glycogen Degradation II	8.35E-05	4/10 (0.4)

Top Upstream Regulators

Upstream Regulator	p-value of overlap	Predicted Activation State
rosiglitazone	8.69E-21	Inhibited
PPARGC1A	1.87E-20	Inhibited
MEF2C	2.41E-19	Inhibited
DMD	8.88E-17	
D-glucose	1.48E-16	

(c) 2000-2014 QIAGEN. All rights reserved.

Top Diseases and Bio Functions

Diseases and Disorders

Name	p-value	# Molecules
Cardiovascular Disease	5.71E-26 - 6.74E-04	138
Organismal Injury and Abnormalities	5.71E-26 - 6.74E-04	307
Developmental Disorder	2.46E-19 - 6.74E-04	84
Skeletal and Muscular Disorders	2.33E-16 - 6.74E-04	109
Hereditary Disorder	2.89E-15 - 6.74E-04	78

Molecular and Cellular Functions

Name	p-value	# Molecules
Cell Morphology	2.97E-18 - 6.74E-04	129
Cellular Development	3.67E-14 - 6.74E-04	171
Cellular Growth and Proliferation	3.67E-14 - 5.41E-04	176
Cellular Assembly and Organization	6.17E-12 - 6.74E-04	121
Cell Death and Survival	4.36E-11 - 6.34E-04	172

(c) 2000-2014 QIAGEN. All rights reserved.

Physiological System Development and Function

Name	p-value	# Molecules
Cardiovascular System Development and Function	6.00E-20 - 6.59E-04	143
Organ Morphology	6.00E-20 - 5.76E-04	115
Skeletal and Muscular System Development and Function	2.97E-18 - 6.74E-04	126
Tissue Morphology	2.97E-18 - 6.74E-04	92
Embryonic Development	3.01E-17 - 4.86E-04	130

(c) 2000-2014 QIAGEN. All rights reserved.

Top Tox Functions

Assays: Clinical Chemistry and Hematology

Name	p-value	# Molecules
Increased Levels of Hematocrit	9.48E-04 - 9.48E-04	9
Increased Levels of Red Blood Cells	1.47E-02 - 1.47E-02	7
Increased Levels of Albumin	5.13E-02 - 5.13E-02	1
Increased Levels of Alkaline Phosphatase	2.60E-01 - 2.60E-01	3
Increased Levels of Creatinine	3.08E-01 - 3.08E-01	1

Cardiotoxicity

Name	p-value	# Molecules
Cardiac Hypertrophy	2.46E-19 - 4.40E-01	48
Cardiac Fibrosis	2.78E-11 - 7.59E-02	25
Heart Failure	5.86E-08 - 5.92E-01	25
Cardiac Arrythmia	1.23E-07 - 4.82E-01	21
Cardiac Necrosis/Cell Death	2.81E-07 - 1.00E-01	25

(c) 2000-2014 QIAGEN. All rights reserved.

Hepatotoxicity

Name	p-value	# Molecules
Liver Steatosis	7.73E-09 - 4.82E-01	27
Liver Necrosis/Cell Death	2.75E-05 - 4.58E-02	19
Liver Hyperplasia/Hyperproliferation	3.89E-05 - 4.54E-01	63
Liver Proliferation	1.01E-04 - 3.78E-01	17
Hepatocellular Carcinoma	8.64E-03 - 4.54E-01	21

Nephrotoxicity

Name	p-value	# Molecules
Renal Damage	5.12E-06 - 5.34E-01	15
Renal Tubule Injury	5.12E-06 - 4.35E-02	15
Kidney Failure	2.65E-03 - 3.65E-01	14
Renal Necrosis/Cell Death	1.02E-02 - 2.50E-01	23
Renal Proliferation	1.30E-02 - 3.61E-01	10

Top Regulator Effect Networks

(c) 2000-2014 QIAGEN. All rights reserved.

ID	Regulators	Diseases & Functions	Consistency Score
1	Calcineurin protein(s), FOXO3, GATA4, HAND2, MEF2A (+8 more)	congestive heart failure (+8 more)	19.132
2	ACOX1, ADIPOQ, FGF1, FST, IL13, PPARA, PPARGC1A (+1 more)	hypoglycemia, quantity of carbohydrate (+1 more)	14.224
3	ACOX1, Akt, DAG1, FGF21, INSR, KLF15, MAP4K4 (+10 more)	damage of muscle, degeneration of heart (+5 more)	13.510
4	Calcineurin protein(s), GATA4, IL1B, MEF2C, MYOCD (+3 more)	congestive heart failure (+1 more)	11.384
5	Calcineurin protein(s), CD24, ESRRA, MEF2A, OXT	congestive heart failure, epithelial neoplasia (+6 more)	7.546

Top Networks

ID	Associated Network Functions	Score
1	Developmental Disorder, Hereditary Disorder, Metabolic Disease	60
2	Energy Production, Nucleic Acid Metabolism, Small Molecule Biochemistry	46
3	Cellular Compromise, Cellular Assembly and Organization, Cell Morphology	44
4	RNA Post-Transcriptional Modification, Gene Expression, Protein Synthesis	41
5	Lipid Metabolism, Nucleic Acid Metabolism, Small Molecule Biochemistry	37

Top Tox Lists

Name	p-value	Ratio
Cardiac Hypertrophy	9.22E-20	48/381 (0.126)
Cardiac Fibrosis	1.07E-11	25/182 (0.137)
Cardiac Necrosis/Cell Death	2.81E-08	25/264 (0.095)
Acute Renal Failure Panel (Rat)	5.01E-07	11/62 (0.177)

(c) 2000-2014 QIAGEN. All rights reserved.

INGENUITY® SYSTEMS

Fatty Acid Metabolism	1.11E-05	13/117 (0.111)

(c) 2000-2014 QIAGEN. All rights reserved.

Top My Lists

Name	p-value	Ratio

Top My Pathways

Name	p-value	Ratio
Network: Gene exp, C.Cycle, conn.Tissue DevNFunct	9.43E-03	2/6 (0.333)

Top Molecules

Fold Change up-regulated

Molecules	Exp. Value	Exp. Chart
THBS1	↑74.844	
LOX	↑61.023	
Pmaip1	↑49.039	
S100A6	↑47.804	
CKS2	↑47.791	
ANXA1	↑47.699	
FOSL1	↑39.380	
EREG	↑37.666	
S100A4	↑37.495	
CXCL2	↑34.666	

Fold Change down-regulated

(c) 2000-2014 QIAGEN. All rights reserved.

Molecules	Exp. Value	Exp. Chart
DSP*	-172.140	
Pln*	-171.472	
MYH6*	-170.245	
SORBS2	-142.896	
TNNI3	-130.703	
CKMT2	-115.025	
ACTC1	-112.428	
MYL2	-107.130	
RYR2*	-106.879	
TNNT2*	-85.146	

(c) 2000-2014 QIAGEN. All rights reserved.

INGENUITY® SYSTEMS
